# Ligand-Based Redox Chemistry and Anti-Kasha Fluorescence in Silver(I) Tripyrrindione Radical

**DOI:** 10.1021/acs.inorgchem.5c05116

**Published:** 2026-02-04

**Authors:** Iva Habenšus, Qi Sun, Andrei V. Astashkin, Lily J. North, Jean-Luc Brédas, Veaceslav Coropceanu, Elisa Tomat

**Affiliations:** † 8041 The University of Arizona, Department of Chemistry and Biochemistry, 1306 E. University Blvd., Tucson, Arizona 85721-0041, United States; ‡ 27258 University of Vienna, Faculty of Chemistry, Institute of Inorganic Chemistry, Währinger Straße 42, 1090 Wien, Austria

## Abstract

Emission from doublet excited states in luminescent radicals enables the design of advantageous properties in optoelectronics and functional materials. Although most investigations focus on polychlorinated triarylmethyl radicals, several other classes of radical emitters are emerging. The tripyrrindione ligand forms a delocalized, luminescent radical when bound to closed-shell ions. Here, we investigate the redox chemistry, coordination, and photophysical properties of tripyrrindione in the presence of the Ag­(I) ion, which is also a widely used oxidant. Two-electron oxidation of the ligand and metal insertion lead to a neutral, diamagnetic complex with T-shaped geometry at the metal center. Subsequent one-electron reduction yields a Ag­(I)-bound tripyrrindione radical as confirmed by crystallographic, electrochemical, spectroscopic, and computational analyses. The air-sensitive, paramagnetic complex exhibits a fluorescence emission band at 653 nm, even though several absorption bands between 750 and 950 nm attest to excited states below the emissive state. Time-dependent DFT calculations attribute this anti-Kasha emission to the radiative decay of the D_3_ state, a feature rationalized by the slow internal conversion of the D_3_ state to the D_2_ state. Given their rich photophysics and ability to stabilize unpaired spins, tripyrrindiones and other oligopyrrolic pigments provide potentially valuable platforms for innovative design of radical emitters.

## Graphical abstract

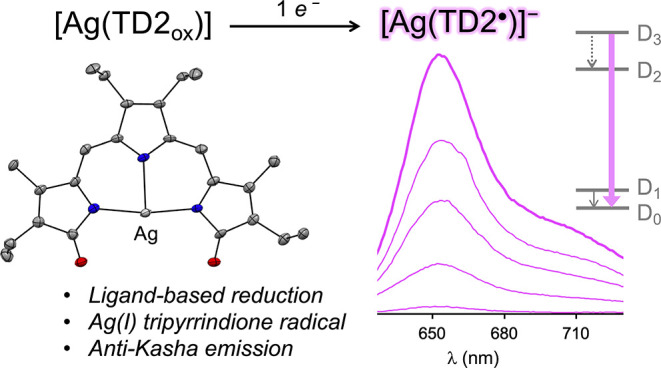

## Introduction

Luminescent radicals are currently the focus of intense research efforts motivated by the potential to access photophysical properties that are not typically attainable with closed-shell molecular emitters.^1,2^ Promising areas of investigations include magnetoluminescence,^3,4^ quantum materials,^5,6^ and high-efficiency organic light-emitting diodes (OLEDs).^7–10^ Defying the common expectation that radicals would be unstable and nonemissive, several classes of robust luminescent radicals have been developed.^1,11^ Polychlorinated triarylmethyl (TArM) radicals (e.g., TTM, Chart 1)^5,7,12,13^ are by far the largest and best studied family of open-shell luminophores; however, additional systems are emerging that include dithiadiazolyl radicals,^9^ *N*-heterocyclic carbene-triphenylamine hybrids,^14^ and 2,5-dimethylpyrrole radical cations.^15^ Among a smaller subgroup of emissive metal-bound radicals, such as gold­(I) complexes of pyridyl-containing TArM radicals^16,17^ and europium­(III) complexes of nitronyl and imino nitroxide radicals,^18,19^ we have previously reported the fluorescence emission of a zinc­(II) complex of the tripyrrindione radical.^20^

**Chart 1:**
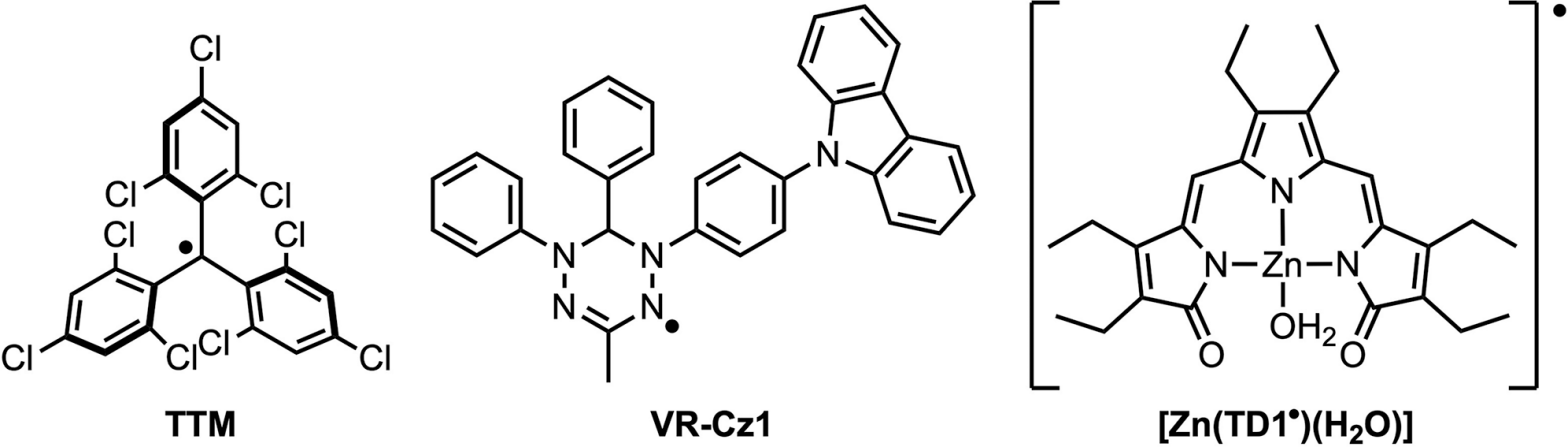
Examples of Luminescent Radicals

The tripyrrindione ligand, which belongs to the family of biopyrrin pigments deriving from heme metabolism,^21^ coordinates several divalent transition metals (e.g., Pd­(II), Pt­(II), Ni­(II), Cu­(II), Zn­(II)) as a tridentate, dianionic radical with spin density delocalized throughout its conjugated π system.^22–25^ Whereas the complexes of *d* ^8^ and *d* ^9^ cations did not show discernible luminescence,^26^ the *d* ^10^ complex [Zn­(TD1^•^)­(H_2_O)] (Chart 1) is emissive at 644 nm upon excitation at 599 nm.^20^ Given the presence of several charge transfer bands between 700 and 950 nm in the absorption spectrum of this complex, the emission appeared to violate Kasha’s rule. An independent computational investigation assigned the anti-Kasha emission to the D_3_ excited state; however, no explanation was proposed for the observed radiative decay.^27^

The exploration of anti-Kasha emission mechanisms offers new opportunities for the application of luminescent radicals.^28^ For instance, whereas emission originating from the D_1_ state is typically in the red to near-infrared region, anti-Kasha emission from higher excited states extends the favorable photophysical properties of luminescent radicals into the visible range. Interesting examples of anti-Kasha emission have been recorded in a variety of stable radicals including triarylmethyl,^29^ verdazyl (e.g., VR-Cz1, Chart 1),^30^ and *N*-heterocyclic carbene systems.^14^

Here, we investigate the coordination of silver in the tripyrrindione framework. We reasoned that coordination of the *d* ^10^ Ag­(I) ion could provide a new route to an emissive tripyrrindione radical. In addition, we sought to examine the interplay of the redox-active tripyrrindione ligand and the redox-active silver cation, which has been employed for the ligand-based oxidation of several tripyrrindione complexes.^22,24,31^ Notably, the oxidation and/or disproportionation of the silver center is also well established in the case of oligopyrrolic ligands: the +2 and/or +3 oxidation states have been documented in silver porphyrins,^32^ carbaporphyrins,^33^ *N*-confused porphyrins,^34^ and corroles.^35–37^ In this study, we describe the preparation of two silver tripyrrindione complexes and their characterization through a combination of spectroscopic, structural, and electrochemical methods. Our computational analysis of the electronic structures provides a rationale for the observed emission properties.

## Results and Discussion

The synthesis of silver­(I) tripyrrindione was achieved by stirring a solution of H_3_TD2 and AgOAc (3 equiv) in DMSO at room temperature under aerobic conditions (Figure 1a). The reaction progress was monitored by UV–visible absorption spectroscopy as the mixture transitioned from the initial orange/red color of the ligand solution to a deep green color of the product, featuring an absorption maximum at 705 nm (Figure 1b). Upon consumption of the ligand, the formation of elemental silver was observed as previously reported for corrole ligands^36^ and likely indicating redox chemistry alongside metal coordination. We found that at least 3.0 equiv of Ag­(I) were required for full conversion to the green complex (Figure S1).

**Figure 1:**
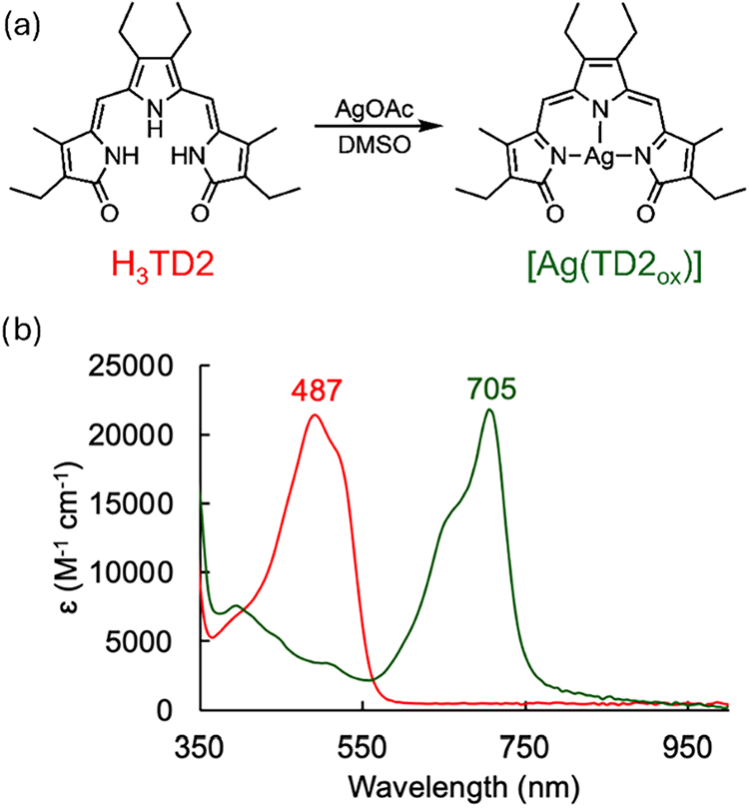
Synthesis of Ag­(I) tripyrrindione [Ag­(TD2_ox_)] (panel a) and UV–visible absorption spectra (panel b) of free ligand H_3_TD2 (red) and complex [Ag­(TD2_ox_)] (green) in CH_2_Cl_2_.

When compared to previously reported tripyrrindione complexes isolated in similar conditions from reactions with acetate salts of divalent ions (e.g., Pd, Cu, Zn),^20,22,24^ this silver complex does not display the long wavelength bands (i.e., 800–1000 nm) characteristic of tripyrrindione radicals. Instead, the band now appearing at 705 nm is most consistent with the absorption of tripyrrindione in its highest oxidation state (i.e., coordinating as a monoanionic tridentate ligand TD2_ox_^–^).^22^ Notably, Ag­(I) was previously employed to oxidize neutral complexes of the tripyrrindione radical (e.g., [Pd­(TD1^•^)­(H_2_O)]) to diamagnetic cationic species (e.g., [Pd­(TD1_ox_)­(H_2_O)]^+^).^22,31^

After purification, the diamagnetic nature of the isolated complex was confirmed by NMR spectroscopy (Figure S2), which indicated the formation of a complex presenting 2-fold symmetry of the tripyrrindione ligand (i.e., two equivalent pyrrolidone moieties) and lacking the aqua ligand found in the fourth coordination site of several square-planar tripyrrindione complexes. The complex was not found to be light-sensitive at ambient conditions; however, slow degradation was observed in the presence of minor acidic impurities in dichloromethane. Chlorinated solvents were therefore passed through a plug of solid NaHCO_3_ prior to all characterization experiments.

Single-crystal X-ray diffraction analysis revealed a neutral silver complex with a flat, tridentate tripyrrindione and a T-shaped coordination geometry (Figures 2 and S3 and Table S1). Consistent with the TD2_ox_^–^ redox state, the bond lengths in the ligand scaffold were similar to those in previously reported [Pt^II^(TD2_ox_)­(H_2_O)]^+^ rather than those in [Pt^II^(TD2^•^)­(H_2_O)] featuring the TD2^2–•^ redox state (Table S2).^31^ Collectively, the spectroscopic and crystallographic data are indicative of Ag­(I) coordination and ligand oxidation rather than disproportionation and coordination of Ag­(II) or Ag­(III) as observed in macrocyclic oligopyrroles.^32–37^

**Figure 2:**
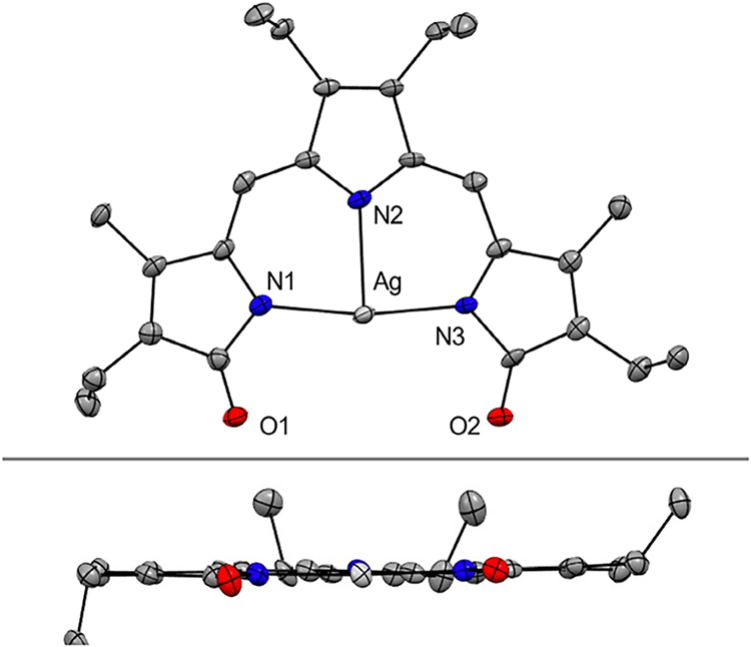
Crystal structure of [Ag­(TD2_ox_)] with partial labeling scheme (top) and side view (bottom). Hydrogen atoms are omitted for clarity. Non-hydrogen atoms are displayed as thermal displacement ellipsoids set at the 50% probability level (CCDC 2350553).

To shed more light on the optical properties of [Ag­(TD2_ox_)], its electronic structure was investigated by density functional theory (DFT) and time-dependent DFT (TD-DFT) methods using the range-separated ωB97X-D functional (see SI). The optimized ground-state geometry shows excellent agreement with the experimental crystal structure data (see Tables S2 and S3), validating the computational approach. The TD-DFT calculations indicate that the first singlet excited state (S_1_) lies at 1.24 eV (1000 nm) over the ground state, and the S_0_ → S_1_ transition displays vanishing oscillator strength (*f* ≈ 0) due to the symmetry-forbidden nature of the transition from the *d*_*x* ^2^–*y* ^2^_ + σ orbital to the π* orbital, as illustrated by the natural transition orbitals (NTOs) in Figure 3. This result explains the absence of long-wavelength absorption bands in the experimental spectrum. In contrast, the second singlet excited state (S_2_) appears at 2.17 eV (571 nm), with the S_0_ → S_2_ transition coupling the π + d_*yz*_ orbital to the π* orbital being symmetry-allowed and exhibiting a significant oscillator strength of *f* = 0.44. The geometry optimization of the S_1_ state yields a transition energy of just 0.13 eV for this state and a zero oscillator strength (*f* = 0), thus explaining why [Ag­(TD2_ox_)] is nonemissive. We note that S_0_ → S_1_ excitation is accompanied by a large reorganization energy of approximately 0.55 eV. This can be attributed to the change in electrostatic energy due to significant modifications (up to 0.24 Å) of the Ag–N bond lengths upon excitation (see Tables S3–S4).

**Figure 3:**
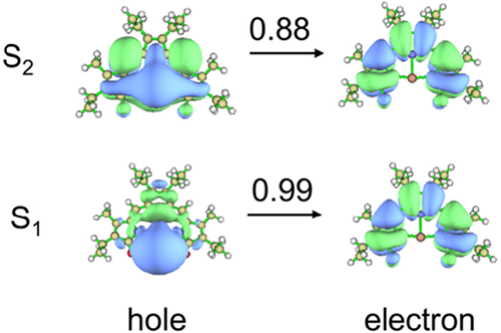
Natural transition orbitals (NTOs) and their weight for the S_0_ → S_1_ and S_0_ → S_2_ transitions in [Ag­(TD2_ox_)] as computed in the ground-state geometry at the TD-DFT/ωB97X-D/def2-SVP level of theory; labels S_1_ and S_2_ indicate the final state of the transition.

The electrochemical profile of [Ag­(TD2_ox_)] was investigated by cyclic voltammetry in CH_2_Cl_2_ (Figure 4a) and presented two quasi-reversible, one-electron reductions at −0.67 and −1.23 V, which were assigned to ligand-based processes based on the known redox chemistry of the tripyrrindione ligand.^22,31^ Additionally, there are two likely metal-centered oxidation events: an irreversible event at 0.37 V attributed to the Ag­(I)/Ag­(II) couple and a quasi-reversible event at 0.64 V for the Ag­(II)/Ag­(III) couple, which is reminiscent of the oxidation of silver­(II) porphyrins.^38^

**Figure 4:**
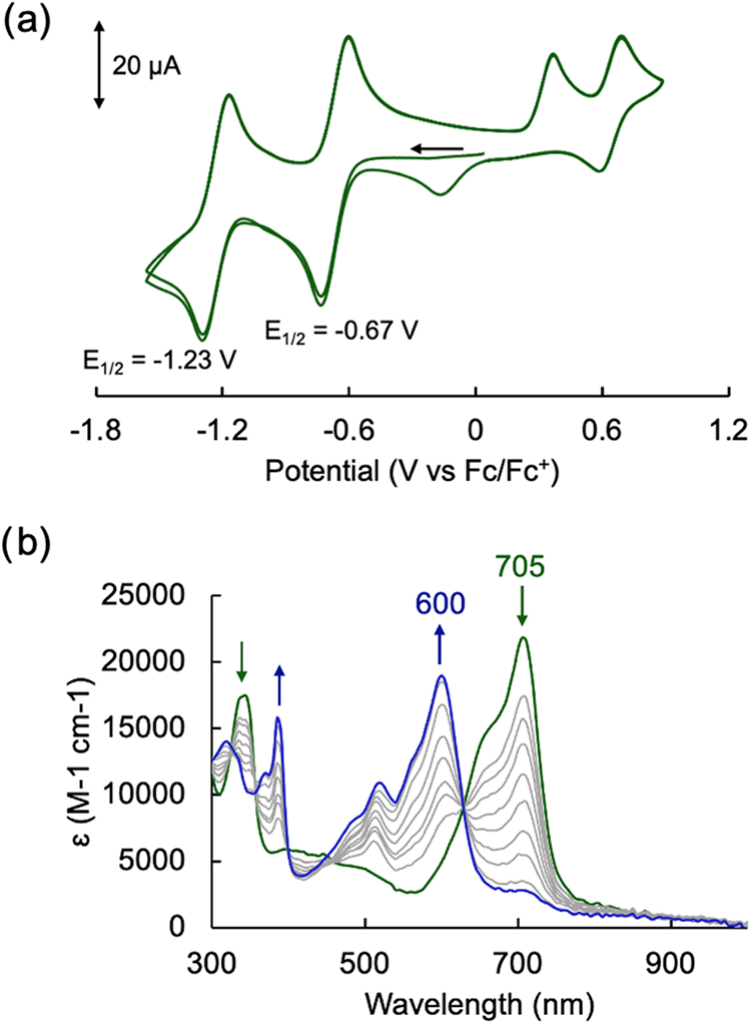
Electrochemical analysis of [Ag­(TD2_ox_)]. (a) Cyclic voltammogram of [Ag­(TD2_ox_)] (1.5 mM) at a glassy carbon electrode in CH_2_Cl_2_ with 0.1 M [(*n*-Bu_4_)­(PF_6_)] as a supporting electrolyte. Data collected at a 100 mV·s^–1^ scan rate using a Ag/AgNO_3_ reference electrode and a Pt wire auxiliary electrode. (b) Spectral changes observed upon reduction of [Ag­(TD2_ox_)] (green trace) (CH_2_Cl_2_, 0.1 M (*n*-Bu_4_)­(PF_6_)) by controlled potential electrolysis (at −1.0 V vs Fc^+^/Fc) to produce the one-electron reduction product [Ag­(TD2^•^)]^−^ (blue trace).

With the goal of characterizing a potentially fluorescent tripyrrindione radical, we further investigated the first reduction event of [Ag­(TD2_ox_)] by controlled-potential electrolysis and monitored the progress via spectroelectrochemical methods (Figure 4b). When the cell potential was held at −1.0 V, the main absorption band at 705 nm decreased while a new band at 600 nm appeared. The new absorption profile with a maximum at 600 nm is consistent with previously reported tripyrrindione radical complexes; however, here we did not observe the well-defined long-wavelength bands associated with intraligand π–π charge transfer in these species.^24^ Because significant broadening of these intraligand bands was previously observed in silver corrole radicals,^39^ we sought to further probe the nature of the reduced species.

Upon reduction of [Ag­(TD2_ox_)] with CoCp_2_ (1 equiv) under inert atmosphere (Scheme 1), an immediate color change from green to blue was observed as well as a pink hue indicative of a fluorescent species. Single crystals grew from a solution of the blue product in CH_2_Cl_2_ layered with pentane at −15 °C. The crystal structure of the reduced species presents an anionic silver complex and a cobaltocenium countercation (Figures 5 and S3). The reduced tripyrrindione complex is similar to the parent complex: the coordination geometry around the Ag­(I) center is again T-shaped and the ligand is relatively planar. Compared to the structure of [Ag­(TD2_ox_)], the largest differences in bond lengths are observed in the ligand scaffold and are consistent with a ligand-based reduction (Table S2). The C–N distances on the pyrrolidone rings are particularly diagnostic of the different oxidation state; however, changes of bond lengths throughout the ligand scaffold reflect the delocalized nature of tripyrrindione radicals.

**Figure 5:**
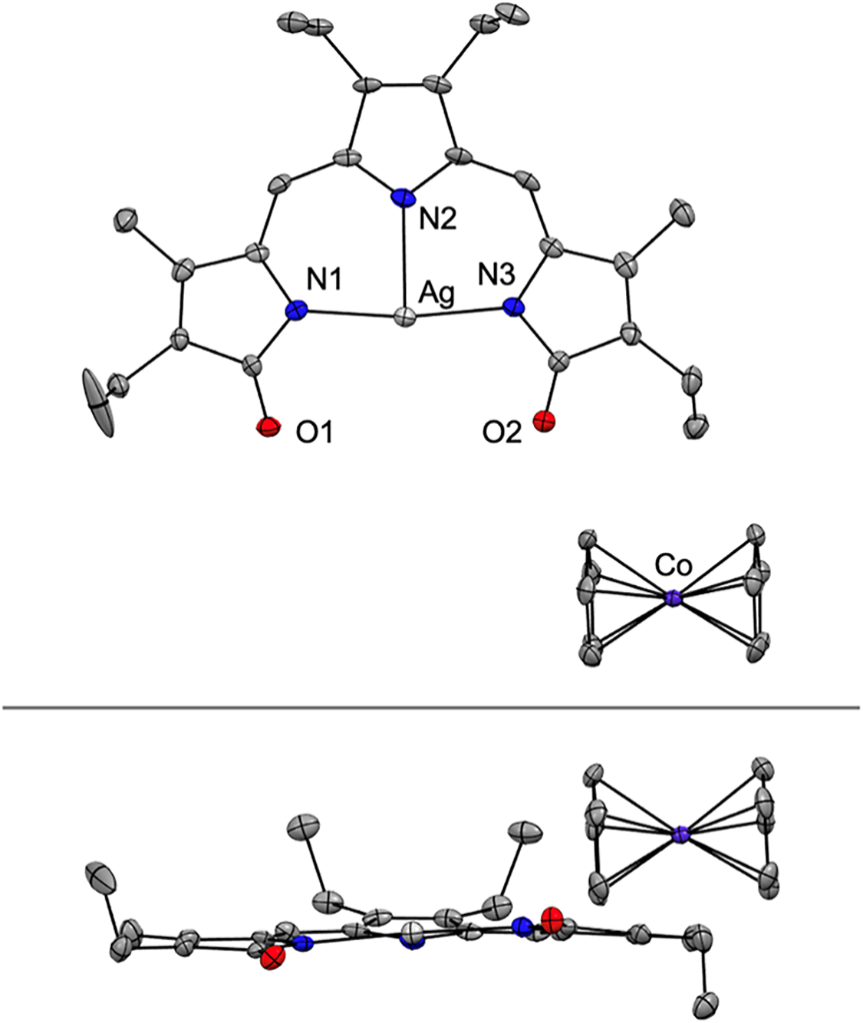
Crystal structure of [CoCp_2_]­[Ag­(TD2^•^)] with partial labeling scheme (top) and side view (bottom). Hydrogen atoms are omitted for clarity. Non-hydrogen atoms are displayed as thermal displacement ellipsoids set at the 50% probability level (CCDC 2350552).

**Scheme 1:**
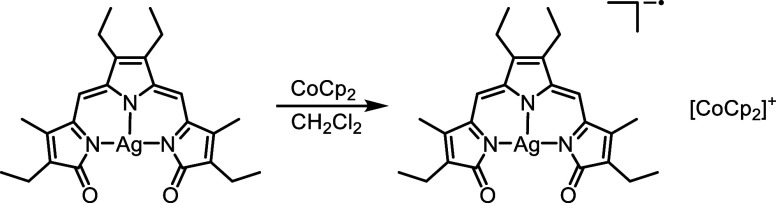
Chemical Reduction of [Ag­(TD2_ox_)] in Dichloromethane to Yield [CoCp_2_]­[Ag­(TD2^•^)]

The reduction to a tripyrrindione radical was also confirmed by EPR spectroscopy. The spectrum of [CoCp_2_]­[Ag­(TD2^•^)] recorded in CH_2_Cl_2_ at room temperature is located at *g* = 2.0029 and exhibits a hyperfine structure with at least 15 lines (Figure 6). The splitting between these lines is about 0.1 mT and the effective width of the spectrum is about 0.5 mT. Interestingly, no hyperfine structure was previously observed for complexes of tripyrrindione radicals.^20,22,24,31^ In earlier experiments, however, the samples of those neutral, air-stable compounds were prepared under aerobic conditions and therefore contained solvated oxygen, which shortens the electronic relaxation times and leads to loss of resolution.^40^ Indeed, when a sample of [Pd­(TD2^•^)­(H_2_O)] was prepared under a nitrogen atmosphere, the hyperfine structure in its room-temperature EPR spectrum became resolved (Figure S4).

**Figure 6:**
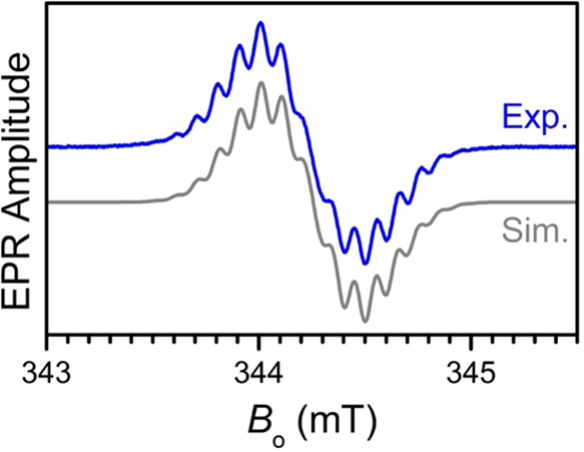
EPR spectrum (blue trace) of [CoCp_2_]­[Ag­(TD2^•^)] (1 mM, CH_2_Cl_2_) at room temperature (298 K); experimental conditions: mw frequency, 9.650 GHz; mw power, 0.2 mW; field modulation amplitude, 0.02 mT. The simulated spectrum (gray trace) was obtained with hfi constants |*a*_N_| = |*a*_H_| = 0.098 mT for one ^14^N and 16 ^1^H nuclei and |*a*_Ag_| = 0.049 mT for the silver center. The individual (Gaussian) line width of 0.075 mT was applied.

The spectrum of the reduced Ag­(I) tripyrrindione (Figure 6) is characteristic of a predominately ligand-centered spin distributed over the ligand π-system, with line splittings caused by the hyperfine interactions (hfi) of the nitrogen nuclei and multiple protons bound to the ligand scaffold or its side chains. Our DFT calculations give a consistent picture, indicating that less than 1% of the spin density is located on the silver center, while the rest is distributed over the ligand. In particular, the calculated spin density distribution here (Figure S5), as well as in earlier DFT calculations for similar systems,^22,41^ show that the central nitrogen (N2) is expected to be the main contributor to the resolved hyperfine structure, whereas the hfi constants of the other two nitrogen nuclei are significantly smaller than the individual line widths of about 0.075 mT. For the two *meso*-protons in α-positions with respect to the radical π-system and 14 protons of the alkyl side chains located in ß-positions with respect to the π-system (taking into account the rapid rotation of these side chains around the C–C bond joining them with the π-system), hfi constants on the order of 0.1 mT are expected.

Numerical simulations show that the experimental EPR spectrum can be reproduced (Figure 6) if the hfi constants of N2 and the 16 protons specified above are taken to be |*a*_N_| = |*a*_H_| = 0.098 mT and, in addition, a hfi constant |*a*_Ag_| = 0.049 mT for the silver nucleus is assumed. Realistically, the values of |*a*_N_| and |*a*_H_| are expected to be somewhat different for different nuclei (except for symmetry-related protons) but still close to 0.098 mT, with their variation contributing to the intrinsic line width of 0.075 mT used in the simulation. The ±0.049 mT hfi constant of the silver nucleus is not resolved; however, it is necessary to achieve an alignment of the resolved hyperfine lines between the experimental and simulated spectra across the whole range. We do not distinguish between the ^109^Ag and ^107^Ag isotopes (which have a natural abundance of ∼50% each and magnetic moments differing by only ∼15%), and the nonzero *a*_Ag_ is indicative of some spin delocalization from the ligand π-system to the d_π_ orbitals of the silver ion. Comparing |*a*_Ag_| ≈ 0.049 mT estimated here with |*a*_Ag_| ≈ 4 mT observed for silver­(II) porphyrin^42,43^ and corrole complexes,^39^ we can estimate the spin population on the silver ion in [Ag­(TD2^•^)]^−^ to be about 1%, which is in line with the DFT results.

Taken collectively, the spectroelectrochemical, crystallographic, and EPR spectroscopic data strongly support a [Ag^I^(TD2^•^)]^−^ configuration, in which the silver center remains in the +1 oxidation state and the spin is localized on the dianionic tripyrrindione radical. This assignment is also consistent with previous observations for related complexes of tripyrrindione radicals (e.g., [Pd­(TD1^•^)­(H_2_O)], [Zn­(TD1^•^)­(H_2_O)], [Pt­(TD2^•^)­(*t*-BuNH_2_)]),^21,25,31,44^ in which the unpaired spin is similarly ligand-centered rather than metal-centered.

The absorption profile of the isolated complex [CoCp_2_]­[Ag­(TD2^•^)] (Figure 7a) displays a maximum at 600 nm and very broad, low-intensity bands between 750 and 1000 nm, likely indicating the presence of a tripyrrindione radical but significantly broader than previously observed.^20,22,24^ The reduced complex is air-sensitive: exposure to air of a dichloromethane solution results in almost complete degradation through partial reoxidation to the parent complex and possibly demetalation over a period of ∼7 min (Figure S6).

**Figure 7:**
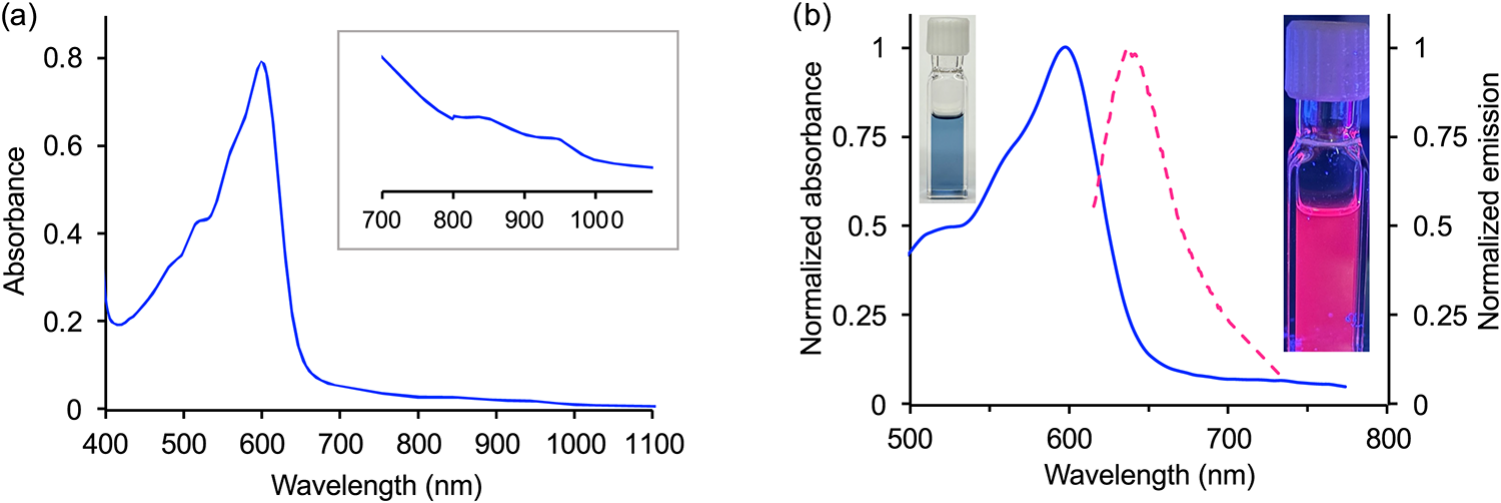
(a) UV–visible absorption spectrum of [CoCp_2_]­[Ag­(TD2^•^)] (42 μM) in CH_2_Cl_2_. (b) Normalized optical absorption (solid blue line) and emission (dashed pink line) traces of [CoCp_2_]­[Ag­(TD2^•^)] in THF at room temperature under a nitrogen atmosphere (λ_ex_ = 605 nm; λ_em_ = 653 nm).

Whereas the formation of a ligand-based radical leads to fluorescence quenching of a borondifluoride dipyrrindione complex,^45^ the reduction of diamagnetic complex [Ag­(TD2_ox_)] and formation of a tripyrrindione radical produces a fluorescent species that was initially observed by the naked eye. When the reduction is carried out stepwise through 0.2 equiv additions of CoCp_2_, the emission intensity (upon excitation at 605 nm) increases proportionally and saturates at 1.0 equiv (Figure S7), consistent with the clean formation of a single fluorescent product rather than a fluorescent impurity or side product. Indeed the isolated product [CoCp_2_]­[Ag­(TD2^•^)] is fluorescent in THF at room temperature under a nitrogen atmosphere: upon excitation at 605 nm, an emission band at 653 nm is observed with a quantum yield (Φ) of 0.11 ± 0.01 (Figure 7b). The excitation spectrum (Figure S8) and the invariance of the emission profile with different excitation wavelengths (from 590 to 610 nm, Figure S9) are indicative of a single emissive species. A concentration study of the emission profile shows no significant self-absorption and only slight shifting at higher concentrations (75–100 μM, Figure S10). When monitored over a period of 3 h, the absorption and emission spectra (Figure S11) indicate slow degradation of the air-sensitive, luminescent species with no interference of emissive impurities.

The photophysical properties of [Ag­(TD2^•^)]^−^ are similar to those of [Zn­(TD1^•^)­(H_2_O)], in which the tripyrrindione radical coordinates a different closed-shell cation and has a slightly higher quantum yield (Φ = 0.23 ± 0.01 for the zinc complex in THF).^20^ In contrast, analogous complexes of the tripyrrindione radical with open-shell metal ions (i.e., [Pd­(TD1^•^)­(H_2_O)], [Cu­(TD1^•^)­(H_2_O)]) do not present discernible fluorescence and likely feature low-lying metal-based excited states facilitating fast, nonradiative relaxation mechanisms.^26,46^

The TD-DFT energies of the [Ag­(TD2^•^)]^−^ anion radical computed at the D_0_ ground-state and D_1_, D_2_, and D_3_ excited-state geometries are summarized in Table 1; the NTOs for selected transitions are shown in Figure 8a (see also Figures S12 and S13). At the ground-state geometry, the first doublet excited state (D_1_) is located at 1.09 eV (1138 nm), and the D_0_ → D_1_ transition has negligible oscillator strength (*f* = 0.0001). As shown in Figure 8a, this transition has a strong metal-to-ligand charge transfer (CT) character and resembles the S_0_ → S_1_ transition of the neutral parent system (Figure 3). The second and third excited doublet states (D_2_ and D_3_) form a pair arising from in-phase and out-of-phase combinations of α- and β-electron π → π* transitions with a negligible metal contribution (see Figure S12). For D_2_, the transition energy is about 1.55 eV (800 nm) with a very small oscillator strength (*f* = 0.0005). In contrast, the D_3_ state located at 2.09 eV (593 nm) is characterized with a much larger oscillator strength of *f* = 0.22. These results correlate well with the experimental absorption spectrum, where a strong absorption maximum observed at ∼600 nm can be attributed to the D_3_ state, while the weak absorption bands spanning 750–1000 nm are associated with the D_1_ and D_2_ states. We note that, given the relatively small energy differences between the excited states, the D_0_ → D_1_ and D_0_ → D_2_ transitions could gain some additional strength via vibronic coupling with the D_3_ state (intensity borrowing mechanism).

**Figure 8:**
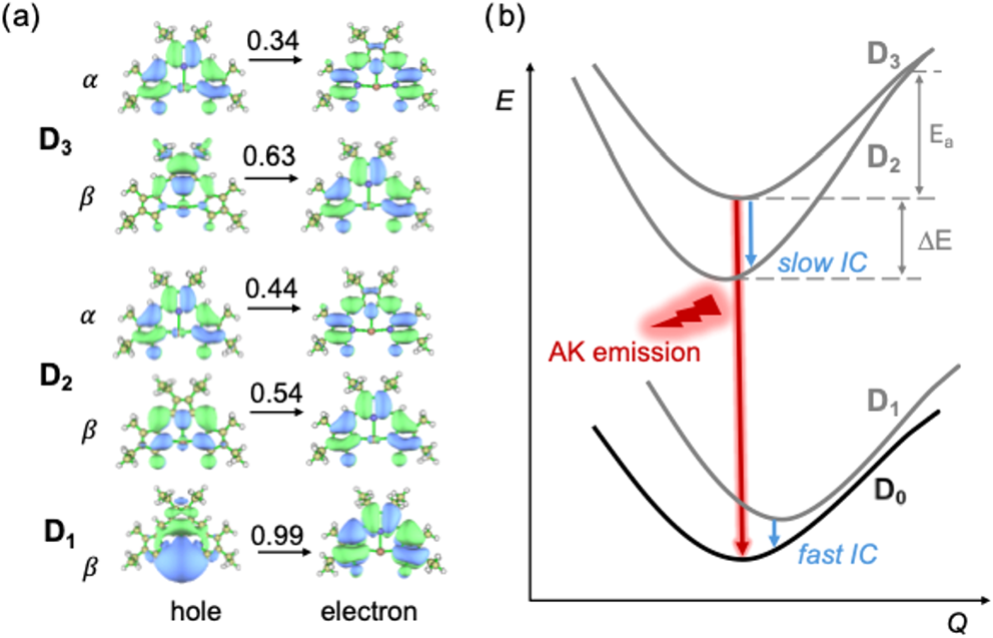
(a) Natural transition orbitals (NTOs) for the D_0_ → D_1_, D_0_ → D_2_, and D_0_ → D_3_ transitions in [Ag­(TD2^•^)]^−^ as computed in the ground-state geometry at the TD-DFT/ωB97X-D/def2-SVP level of theory. The numbers above the arrows denote the weight of the particular NTO in the description of the transition. (b) Schematic illustration of the proposed anti-Kasha (AK) emission mechanism for [Ag­(TD2^•^)]^−^.

**Table 1:** Energies (*E*, in eV) and Corresponding Oscillator Strengths (*f*) of the D_0_ → D_1_, D_0_ → D_2_, and D_0_ → D_3_ Transitions in [Ag­(TD2^•^)]^−^ as Derived at the Ground-State (D_0_) Geometry and D_1_, D_2_, and D_3_ Excited-State Geometries^a^

geometry	D_0_		D_1_		D_2_		D_3_	
state	*E*	*f*	*E*	*f*	*E*	*f*	*E*	*f*
D_1_	1.09	0.0001	0.03	0.0000	1.24	0.0000	1.12	0.0000
D_2_	1.55	0.0005	1.45	0.0005	1.44	0.0253	1.47	0.0044
D_3_	2.09	0.2206	1.68	0.0306	2.06	0.4821	1.98	0.2812

a TD-DFT calculations performed at the ωB97X-D/def2-SVP level.

The optimized structures of the D_2_ and D_3_ states closely resemble that of the ground state, particularly with respect to the Ag–N bond lengths. At the D_1_ geometry, the estimated vertical D_1_ → D_0_ transition energy is merely 0.03 eV, indicating that the potential energy surfaces (PES) of D_0_ and D_1_ are quasi-degenerate (Figure 8b). This feature indicates that D_1_ is expected to undergo a very fast nonradiative relaxation to the ground state. At the optimized D_3_ geometry, the vertical D_3_ → D_0_ transition energy is calculated to be 1.98 eV (626 nm), which is in good agreement with the experimental emission maximum measured at 653 nm and consistent with the anti-Kasha emission observed for this complex. The calculated oscillator strength for D_3_ → D_0_ is *f* = 0.28; according to Einstein’s approach ( $k_{r}\approx \frac{2E_{\mathrm{em}}^{2}f}{3}$ , with the emission energy *E*_em_ given here in cm^–1^),^47^ this yields a radiative rate constant *k*_r_ of 4.7 × 10^7^ s^–1^. Notably, a similar D_3_ state was previously proposed as the origin of anti-Kasha emission on the basis of electronic structure calculations for [Zn­(TD1^•^)­(H_2_O)],^27^ which features the same tripyrrindione electronic configuration of [Ag­(TD2^•^)]^−^.

Based on the properties of PESs derived by means of TD-DFT calculations, we suggest that the emission in [CoCp_2_]­[Ag­(TD2^•^)] as well as in [Zn­(TD1^•^)­(H_2_O)] can be rationalized in terms of a type-I anti-Kasha scenario,^47^ which was first described in the case of azulene^48,49^ and is characterized by large electronic couplings and weak vibrational couplings. Indeed, our TD-DFT calculations show that the energy difference (Δ*E*) between the minima of the PESs of D_2_ and D_3_ is about 0.5 eV while the corresponding reorganization energy (λ) is about 0.06 eV. These values, according to a two-state model (*E*_a_ = (Δ*E* – λ)^2^/4λ), yield an energy of about 0.8 eV for the classical activation energy barrier related to the internal conversion (IC) from the D_3_ state to the D_2_ state (see Figure 8b). Such a large activation energy is expected to lead to slow nonradiative decay of D_3_, thereby opening up the route for the observed anti-Kasha emission. The value estimated above for *k*_r_, taken together with the experimental value for the quantum yield (Φ = 0.11), suggests that the D_3_→D_2_ IC rate constant is not exceeding 5.0 × 10^8^ s^–1^.

Although insights on the anti-Kasha emission of luminescent radicals are still emerging,^28^ the fluorescent tripyrrindione radicals appear structurally and mechanistically distinct. For instance, in several cases, including dioxoborocyclic radicals^50^ and the photogenerated azaxanthone ketyl radical,^51^ the anti-Kasha emission is attributed to large energy gaps (e.g., close to 1 eV or higher) and therefore suppressed IC between higher excited states and the first excited state (D_1_). A full understanding of the parameters governing the emission from higher doublet states, however, will require further spectroscopic investigations as well as the preparation of related analogs designed to explore the photophysical profile of these compounds.

## Conclusion

The tripyrrindione ligand has been previously incorporated in complexes of square planar and square pyramidal geometries featuring divalent and trivalent transition metals. Here, we showed that treatment with Ag­(I) leads to two-electron oxidation of the ligand and formation of the Ag­(I) complex [Ag­(TD2_ox_)], which has a T-shaped geometry at the metal center and a monoanionic, planar tripyrrindione framework. Electrochemical and computational findings indicate that this neutral complex undergoes reversible one-electron reduction of the ligand system to produce the anionic Ag­(I) complex of the tripyrrindione radical. Upon chemical reduction of [Ag­(TD2_ox_)] with cobaltocene, [CoCp_2_]­[Ag­(TD2^•^)] was isolated, and its EPR characterization confirmed a delocalized, TD2-based radical. In spite of the different synthetic route and geometry at the metal center, the anionic complex [Ag­(TD2^•^)]^−^ recapitulates the electronic configuration of [Zn­(TD1^•^)­(H_2_O)], in which a tripyrrindione radical is bound to a *d* ^10^ metal.

Unlike TD1 and TD2 radical complexes of open-shell metal ions, these *d* ^10^ complexes exhibit a bright fluorescence that was initially detected by the naked eye in the synthetic laboratory. The fluorescence emission of [Ag­(TD2^•^)]^−^ at 653 nm, significantly blue-shifted relative to the weak absorption bands between 750 and 1000 nm, is attributed to the D_3_ excited state and therefore to an anti-Kasha mechanism. Our TD-DFT analysis indicates that a high activation energy and therefore slow internal conversion from the D_3_ to the D_2_ state allow for radiative decay from the D_3_ state. A full spectroscopic investigation is underway to further characterize the relaxation dynamics of the emissive tripyrrindione radical in *d* ^10^ complexes.

## Experimental Section

### Materials and Methods

H_3_TD2 was synthesized according to a previously reported procedure.^31^ Dichloromethane (CH_2_Cl_2_) and pentane were dried by passage through a Vacuum Atmospheres solvent purifier. Dry solvents were confirmed to contain <0.1 ppm of H_2_O using a Mettler Toledo C10S Coulometric Karl Fisher Titrator. All other commercial reagents were used without further purification. ^1^H and ^13^C NMR spectra were recorded on a Bruker NEO-500 instrument at the NMR Spectroscopy Facility of the University of Arizona Department of Chemistry and Biochemistry (RRID:SCR_012716). UV–visible absorption spectra were obtained at ambient temperature using an Agilent Cary 60 spectrophotometer. Elemental analyses were performed by Numega Resonance Laboratories, San Diego, CA. Low- and high-resolution mass spectra (LRMS and HRMS) via electrospray ionization (ESI) methods were obtained at the University of Arizona Analytical & Biological Mass Spectrometry Core Facility (RRID:SCR_023370). The X-band EPR measurements were performed at the University of Arizona EPR Facility (RRID:SCR_022883) on a continuous-wave Elexsys E500 spectrometer (Bruker Biospin) equipped with a rectangular TE_102_ resonator.

### Synthesis of [Ag­(TD2_ox_)]

H_3_TD2 (30 mg, 0.076 mmol) was dissolved in DMSO (1 mL) and AgOAc (38 mg, 0.23 mmol) was added to the flask. After stirring for 10 min, the mixture was diluted with ethyl acetate (25 mL) and washed with water (3 × 25 mL) and brine (3 × 25 mL). The organic layer was dried over anhydrous sodium sulfate and concentrated down to 1–2 mL. The product precipitated upon addition of pentane to yield a green solid (16 mg, 42% yield). UV–Vis (CH_2_Cl_2_) λ_max_ (ε) 340 (18,700), 659 (15,500), 705 (21,800 M^–1^ cm^–1^). ^1^H NMR (500 MHz, CDCl_3_): δ 6.05 (s, 2H), 2.57 (q, *J* = 7.6 Hz, 4H), 2.40 (q, *J* = 7.6 Hz, 4H), 2.15 (s, 6H), 1.21 (t, *J* = 7.6 Hz, 6H), 1.12 (t, *J* = 7.6 Hz, 6H). ^13^C NMR (125 MHz, CDCl_3_): δ 184.7, 178.2, 173.5, 147.5, 146.3, 138.4, 96.6, 18.1, 17.7, 15.9, 13.6, 10.7. HRMS-ESI^+^ (*m*/*z*): [M + H]^+^ calcd. for [C_24_H_29_AgN_3_O_2_], 498.13052 (100%), 500.13018 (93%); found, 498.13091 (100%), 500.13030 (95%). Anal. Calcd. for [C_24_H_28_N_3_O_2_Ag]: C, 57.8; H, 5.7; N, 8.4%; found: C, 57.4; H, 5.3; N, 8.3%.

### Synthesis of [CoCp_2_]­[Ag­(TD2^•^)]

In a nitrogen atmosphere inside a glovebox, [Ag­(TD2_ox_)] (10 mg, 0.021 mmol) was dissolved in CH_2_Cl_2_ (1 mL) and CoCp_2_ (4 mg, 0.021 mmol) was added. An immediate color change from green to blue was observed. The sample was filtered through a glass wool plug and precipitated with pentane to yield a dark blue solid (8 mg, 55% yield). UV–Vis (CH_2_Cl_2_; determined from spectroelectrochemical data) λ_max_ (ε) 385 (15,800), 600 (19,000 M^–1^ cm^–1^). Anal. Calcd. for [C_34_H_38_N_3_O_2_AgCo]·0.5CH_2_Cl_2_: C, 56.8; H, 5.4; N, 5.8%; found: C, 56.6; H, 5.5; N, 5.8%.

### X-ray Diffraction Analysis

Single-crystal X-ray diffraction measurements were performed at the University of Arizona XRD Facility (RRID:SCR_022886) on a Bruker D8 Venture instrument equipped with Mo IμS 3.0 microsource and Photon 3 detector. The measurement temperature was 100 K. The absorption correction was done using a multiscan method in *SADABS* (Sheldrick G. M. University of Göttingen, Germany, 1997). The structures were solved and refined with the SHELX package^52^ accessed from the Olex2 ^53,54^ graphic environment. All non-H atoms were located in the Fourier map and refined anisotropically. Carbon-bound hydrogen atoms were calculated in ideal positions, with isotropic displacement parameters set to 1.2*U*_eq_ of the host atom (1.5*U*_eq_ for methyl hydrogen atoms). Their positions were then refined using a riding model. The details pertaining to the experiment and to structure refinement are available in Table S1.

#### Structure Refinement of [Ag­(TD2_ox_)]

Crystals grew as dark green plates by slow diffusion of hexane in a solution of the complex in ethyl acetate. Data collection was optimized for the monoclinic system, and the structure was solved and refined in the monoclinic space group *C*2/*c*. The asymmetric unit cell contained one metal complex. The highest residual Fourier peak found in the model was +1.27 e Å^–3^ approximately 1.06 Å from N1 and the deepest Fourier hole was −0.99 e Å^–3^ approximately 1.58 Å from H22A. The small crystal size (Table S1) resulted in reduced data quality, large *R*_int_ value (∼0.2), and a corresponding B-level alert in the CheckCIF report. There were no ambiguities, however, in the structure solution/refinement.

#### Structure Refinement of [CoCp_2_]­[Ag­(TD2^•^)]

Crystals grew as dark blue blocks by slow diffusion of hexane in a solution of the complex in CH_2_Cl_2_ at −15 °C under a nitrogen atmosphere. Data collection was optimized for the triclinic system and the structure was solved and refined in the triclinic space group *P*1̅. The asymmetric unit cell contained one metal complex. The highest residual Fourier peak found in the model was +1.27 e Å^–3^ approximately 1.06 Å from N1 and the deepest Fourier hole was −0.99 e Å^–3^ approximately 1.58 Å from H22A.

### Electrochemical Measurements

Cyclic voltammograms were performed on a Gamry Reference 600 potentiostat utilizing a single-compartment cell with three electrodes: a glassy carbon working electrode, a platinum wire auxiliary electrode, and a Ag/AgCl reference electrode. Measurements were performed at ambient temperature under an inert argon atmosphere in CH_2_Cl_2_ containing 0.1 M [(*n*-Bu_4_N)­(PF_6_)] (triply recrystallized) as a supporting electrolyte. Sample concentrations were 1–2 mM and all electrochemical data were internally referenced to the ferrocene/ferrocenium couple (set at 0.00 V).

### Fluorescence Measurements

Fluorescence spectra were recorded on a PTI QuantaMaster 400 Steady-State Spectrofluorometer using PTI FelixGX software. Fluorescence quantum yields were calculated with the equation below using rhodamine 6G as a standard reference (λ_ex_ = 530 nm; λ_em_ = 551 nm)

$$\Phi_{S}=\Phi_{\mathrm{ref}}\left(\frac{I_{S}}{I_{\mathrm{ref}}}\right)\left(\frac{A_{\mathrm{ref}}}{A_{S}}\right){\left(\frac{\eta_{S}}{\eta_{\mathrm{ref}}}\right)}^{2}$$

where Φ_S_ and Φ_ref_ are the quantum yields of the sample and the rhodamine 6G reference (Φ_ref_ = 0.95 in EtOH),^55^ respectively, *I*_S_ and *I*_ref_ are the integrated emission intensities, *A*_S_ and *A*_ref_ are the absorbance values at the excitation wavelengths, and η_S_ and η_ref_ are the refractive indices of the solvents used. Measurements were conducted under ambient conditions for rhodamine 6G and under inert conditions for [CoCp_2_]­[Ag­(TD2^•^)] using a 0.5 cm × 0.5 cm cuvette and 1.00 mm slit widths.

### Computational Details

Geometry optimizations of the closed-shell [Ag­(TD2_ox_)] and open-shell [Ag­(TD2^•^)]^−^ complexes were performed using density functional theory (DFT) and unrestricted DFT (U-DFT), respectively. Excited-state geometry optimizations were carried out with the unrestricted time-dependent DFT (TD-DFT) method. For the D1 and D2 excited-state structures, the optimizations were conducted with the Q-Chem package.^56^ All geometry optimizations employed the ωB97X-D functional in combination with the 6–31G* basis set. To describe the excited-state properties more accurately, system-specific range-separation parameters (ω) were applied: ω = 0.1557 for [Ag­(TD2_ox_)] and ω = 0.0501 for [Ag­(TD2^•^)]^−^. Solvent effects were included using the polarizable continuum model (PCM) with dichloromethane as the implicit solvent. Unless otherwise specified, all computations were performed with the Gaussian 16 software package (Revision B.01; Gaussian, Inc., Wallingford CT: 2016).^57^ Natural transition orbital (NTO) analyses and orbital visualizations were carried out using the Multiwfn software,^58,59^ while reorganization energies were obtained from normal-mode frequency calculations.^60^

## Supplementary Material



## Notes

Experimental and computational details, characterization data (PDF)

## References

[1] Mizuno A., Matsuoka R., Mibu T., Kusamoto T. (2024). Luminescent Radicals. Chem. Rev..

[2] Ji L., Shi J., Wei J., Yu T., Huang W. (2020). Air-Stable
Organic
Radicals: New-Generation Materials for Flexible Electronics?. Adv. Mater..

[3] Kimura S., Kusamoto T., Kimura S., Kato K., Teki Y., Nishihara H. (2018). Magnetoluminescence
in a Photostable, Brightly Luminescent
Organic Radical in a Rigid Environment. Angew.
Chem., Int. Ed..

[4] Mizuno A., Matsuoka R., Kimura S., Ochiai K., Kusamoto T. (2024). Spin-Correlated
Luminescence of a Carbazole-Containing Diradical Emitter: Single-Molecule
Magnetoluminescence and Thermally Activated Emission. J. Am. Chem. Soc..

[5] Gorgon S., Lv K., Grüne J., Drummond B. H., Myers W. K., Londi G., Ricci G., Valverde D., Tonnelé C., Murto P. (2023). Reversible spin-optical interface in luminescent organic
radicals. Nature.

[6] Poh Y. R., Morozov D., Kazmierczak N. P., Hadt R. G., Groenhof G., Yuen-Zhou J. (2024). Alternant
Hydrocarbon Diradicals as Optically Addressable
Molecular Qubits. J. Am. Chem. Soc..

[7] Ai X., Evans E. W., Dong S., Gillett A. J., Guo H., Chen Y., Hele T. J. H., Friend R. H., Li F. (2018). Efficient
radical-based light-emitting diodes with doublet emission. Nature.

[8] Peng Q., Obolda A., Zhang M., Li F. (2015). Organic Light-Emitting
Diodes Using a Neutral π Radical as Emitter: The Emission from
a Doublet. Angew. Chem., Int. Ed..

[9] Beldjoudi Y., Nascimento M. A., Cho Y. J., Yu H., Aziz H., Tonouchi D., Eguchi K., Matsushita M. M., Awaga K., Osorio-Roman I. (2018). Multifunctional Dithiadiazolyl
Radicals: Fluorescence, Electroluminescence, and Photoconducting Behavior
in Pyren-1′-yl-dithiadiazolyl. J. Am.
Chem. Soc..

[10] Lu C., Cho E., Wan K., Wu C., Gao Y., Coropceanu V., Brédas J.-L., Li F. (2024). Achieving Nearly 100% Photoluminescence
Quantum Efficiency in Organic Radical Emitters by Fine-Tuning the
Effective Donor-Acceptor Distance. Adv. Funct.
Mater..

[11] Chen Z. X., Li Y., Huang F. (2021). Persistent and Stable
Organic Radicals: Design, Synthesis,
and Applications. Chem.

[12] Cho E., Coropceanu V., Brédas J.-L. (2020). Organic Neutral Radical Emitters:
Impact of Chemical Substitution and Electronic-State Hybridization
on the Luminescence Properties. J. Am. Chem.
Soc..

[13] Cho E., Sun Q., McBride E. P., Brédas J.-L., Coropceanu V. (2025). Luminescent
Radicals Based on the Tris­(trichlorophenyl)­methyl acceptor: How to
Choose the Donor Component. ACS Mater. Lett..

[14] Li X., Wang Y.-L., Chen C., Han Y.-F. (2023). Luminescent Crystalline
Carbon- and Nitrogen-Centered Organic Radicals Based on N-Heterocyclic
Carbene-Triphenylamine Hybrids. Chem. - Eur.
J..

[15] Zhou Z., Qian J., Liu K., Zhang Y., Gao M., Tang B. Z. (2022). A Water-Stable and Red-Emissive Radical Cation for
Mutp53 Cancer Therapy. Angew. Chem., Int. Ed..

[16] Hattori Y., Kusamoto T., Nishihara H. (2015). Enhanced Luminescent
Properties of
an Open-Shell (3,5-Dichloro-4-pyridyl)­bis­(2,4,6-trichlorophenyl)­methyl
Radical by Coordination to Gold. Angew. Chem.,
Int. Ed..

[17] Ogino Y., Kusamoto T., Hattori Y., Shimada M., Tsuchiya M., Yamanoi Y., Nishibori E., Sugimoto K., Nishihara H. (2017). Solvent-Controlled
Doublet Emission of an Organometallic Gold­(I) Complex with a Polychlorinated
Diphenyl­(4-pyridyl)­methyl Radical Ligand: Dual Fluorescence and Enhanced
Emission Efficiency. Inorg. Chem..

[18] Lescop C., Luneau D., Rey P., Bussière G., Reber C. (2002). Synthesis, Structures, and Magnetic
and Optical Properties of a Series
of Europium­(III) and Gadolinium­(III) Complexes with Chelating Nitronyl
and Imino Nitroxide Free Radicals. Inorg. Chem..

[19] Lannes A., Intissar M., Suffren Y., Reber C., Luneau D. (2014). Terbium­(III)
and Yttrium­(III) Complexes with Pyridine-Substituted Nitronyl Nitroxide
Radical and Different β-Diketonate Ligands. Crystal Structures
and Magnetic and Luminescence Properties. Inorg.
Chem..

[20] Gautam R., Petritis S. J., Astashkin A. V., Tomat E. (2018). Paramagnetism and Fluorescence
of Zinc­(II) Tripyrrindione: A Luminescent Radical Based on a Redox-Active
Biopyrrin. Inorg. Chem..

[21] Tomat E., Curtis C. J. (2021). Biopyrrin Pigments:
From Heme Metabolites to Redox-Active
Ligands and Luminescent Radicals. Acc. Chem.
Res..

[22] Gautam R., Loughrey J. J., Astashkin A. V., Shearer J., Tomat E. (2015). Tripyrrindione
as a Redox-Active Ligand: Palladium­(II) Coordination in Three Redox
States. Angew. Chem., Int. Ed..

[23] Bahnmüller S., Plotzitzka J., Baabe D., Cordes B., Menzel D., Schartz K., Schweyen P., Wicht R., Bröring M. (2016). Hexaethyltripyrrindione
(H3Et6tpd): A Non-Innocent Ligand Forming Stable Radical Complexes
with Divalent Transition-Metal Ions. Eur. J.
Inorg. Chem..

[24] Gautam R., Astashkin A. V., Chang T. M., Shearer J., Tomat E. (2017). Interactions
of Metal-Based and Ligand-Based Electronic Spins in Neutral Tripyrrindione
π Dimers. Inorg. Chem..

[25] Habenšus I., Ghavam A., Curtis C. J., Astashkin A. V., Tomat E. (2023). Primary amines as ligands and linkers
in complexes of tripyrrindione
radicals. J. Porphyrins Phthalocyanines.

[26] Cho B., Swain A., Gautam R., Tomat E., Huxter V. M. (2022). Time-resolved
dynamics of stable open- and closed-shell neutral radical and oxidized
tripyrrindione complexes. Phys. Chem. Chem.
Phys..

[27] Heil A., Marian C. M. (2019). DFT/MRCI-R2018 study
of the photophysics of the zinc­(ii)
tripyrrindione radical: non-Kasha emission?. Phys. Chem. Chem. Phys..

[28] Zhu Y., Zhu Z., Wang S., Kuang Z., Peng Q., Abdurahman A. (2025). Anti-Kasha
Emission in Organic Radicals: Mechanistic Insights and Experimental
Caveats. ChemPhotoChem.

[29] Zhu Z., Kuang Z., Shen L., Wang S., Ai X., Abdurahman A., Peng Q. (2024). Dual Channel Emissions of Kasha and
Anti-Kasha from a Single Radical Molecule. Angew.
Chem., Int. Ed..

[30] Gao S., Ding J., Yu S., Li F. (2023). Stable nitrogen-centered
radicals with anti-Kasha emission. J. Mater.
Chem. C.

[31] Tomat E., Curtis C. J., Astashkin A. V., Conradie J., Ghosh A. (2023). Multicenter
interactions and ligand field effects in platinum­(II) tripyrrindione
radicals. Dalton Trans..

[32] Scheidt W. R., Mondal J. U., Eigenbrot C. W., Adler A., Radonovich L. J., Hoard J. L. (1986). Crystal and molecular
structure of the silver­(II) and
zinc­(II) derivatives of meso-tetraphenylporphyrin. An exploration
of crystal-packing effects on bond distance. Inorg. Chem..

[33] Muckey M. A., Szczepura L. F., Ferrence G. M., Lash T. D. (2002). Silver­(III) Carbaporphyrins:
The First Organometallic Complexes of True Carbaporphyrins. Inorg. Chem..

[34] Furuta H., Ogawa T., Uwatoko Y., Araki K. (1999). N-Confused Tetraphenylporphyrin–Silver­(III)
Complex. Inorg. Chem..

[35] Brückner C. (2004). The Silver
Complexes of Porphyrins, Corroles, and Carbaporphyrins: Silver in
the Oxidation States II and III. J. Chem. Educ..

[36] Brückner C., Barta C. A., Briñas R. P., Krause Bauer J. A. (2003). Synthesis
and Structure of [meso-Triarylcorrolato]­silver­(III). Inorg. Chem..

[37] Thomas K. E., Vazquez-Lima H., Fang Y., Song Y., Gagnon K. J., Beavers C. M., Kadish K. M., Ghosh A. (2015). Ligand Noninnocence
in Coinage Metal Corroles: A Silver Knife-Edge. Chem. - Eur. J..

[38] Kadish K. M., Lin X. Q., Ding J. Q., Wu Y. T., Araullo C. (1986). A reinvestigation
of silver porphyrin electrochemistry. Reactions of silver­(III), silver­(II),
and silver­(I). Inorg. Chem..

[39] Sinha W., Sommer M. G., Deibel N., Ehret F., Sarkar B., Kar S. (2014). Silver Corrole Complexes:
Unusual Oxidation States and Near-IR-Absorbing
Dyes. Chem. - Eur. J..

[40] Hyde, J. S. ; Subczynski, W. K. Spin-Label Oxymetry. In Biological Magnetic Resonance; Berliner, L. J. ; Reuben, J. , Eds.; Plenum Press, 1989; Vol. 8, pp 399–425.

[41] Curtis C. J., Habenšus I., Conradie J., Bardin A. A., Nannenga B. L., Ghosh A., Tomat E. (2024). Gold Tripyrrindione: Redox Chemistry
and Reactivity with Dichloromethane. Inorg.
Chem..

[42] Fukuzumi S., Ohkubo K., Zhu W., Sintic M., Khoury T., Sintic P. J., E W., Ou Z., Crossley M. J., Kadish K. M. (2008). Androgynous Porphyrins. Silver­(II) Quinoxalinoporphyrins
Act as Both Good Electron Donors and Acceptors. J. Am. Chem. Soc..

[43] Ghazzali M., Abu-Youssef M. A. M., Larsson K., Hansson Ö., Amer A., Tamm T., Öhrström L. (2008). Synthesis, EPR and
DFT calculations of rare Ag­(II)­porphyrins and the crystal structure
of [Zn­(II)­tetrakis­(4-bromo-2-thiophene)­porphyrin]. Inorg. Chem. Commun..

[44] Habenšus I., Tomat E. (2024). meso-Aryl substituents modify the
electrochemical profile and palladium­(II)
coordination of redox-active tripyrrin ligands. Inorg. Chem. Front..

[45] Gautam R., Petritis S. J., Tomat E. (2019). Redox-Switchable Cyan Fluorescence
of a BODIPY Analog Inspired by Propentdyopent Pigments. Eur. J. Inorg. Chem..

[46] Kumar A., Thompson B., Gautam R., Tomat E., Huxter V. (2023). Temperature-Dependent
Spin-Driven Dimerization Determines the Ultrafast Dynamics of a Copper­(II)-Bound
Tripyrrindione Radical. J. Phys. Chem. Lett..

[47] Veys K., Escudero D. (2022). Anti-Kasha Fluorescence
in Molecular Entities: Central
Role of Electron–Vibrational Coupling. Acc. Chem. Res..

[48] Viswanath G., Kasha M. (1956). Confirmation of the Anomalous Fluorescence
of Azulene. J. Chem. Phys..

[49] Veys K., Escudero D. (2020). Computational Protocol
To Predict Anti-Kasha Emissions:
The Case of Azulene Derivatives. J. Phys. Chem.
A.

[50] Feng Z., Chong Y., Tang S., Ruan H., Fang Y., Zhao Y., Jiang J., Wang X. (2021). Stable Boron-Containing
Blue-Photoluminescent Radicals. Chin. J. Chem..

[51] Sakamoto M., Cai X., Hara M., Tojo S., Fujitsuka M., Majima T. (2005). Anomalous Fluorescence
from the Azaxanthone Ketyl Radical
in the Excited State. J. Am. Chem. Soc..

[52] Sheldrick G. M. (2015). SHELXT
- Integrated space-group and crystal-structure determination. Acta Crystallogr., Sect. A: Found. Adv..

[53] Dolomanov O. V., Bourhis L. J., Gildea R. J., Howard J. A. K., Puschmann H. (2009). OLEX2: a complete
structure solution, refinement and analysis program. J. Appl. Crystallogr..

[54] Bourhis L. J., Dolomanov O. V., Gildea R. J., Howard J. A. K., Puschmann H. (2015). The anatomy
of a comprehensive constrained, restrained refinement program for
the modern computing environment – Olex2 dissected. Acta Crystallogr., Sect. A: Found. Adv..

[55] Magde D., Wong R., Seybold P. G. (2002). Fluorescence
Quantum Yields and Their
Relation to Lifetimes of Rhodamine 6G and Fluorescein in Nine Solvents:
Improved Absolute Standards for Quantum Yields. Photochem. Photobiol..

[56] Epifanovsky E., Gilbert A. T. B., Feng X., Lee J., Mao Y., Mardirossian N., Pokhilko P., White A. F., Coons M. P., Dempwolff A. L. (2021). Software for the frontiers
of quantum chemistry:
An overview of developments in the Q-Chem 5 package. J. Chem. Phys..

[57] Frisch, M. J. ; Trucks, G. W. ; Schlegel, H. B. ; Scuseria, G. E. ; Robb, M. A. ; Cheeseman, J. R. ; Scalmani, G. ; Barone, V. ; Petersson, G. A. ; Nakatsuji, H. Gaussian 16, Rev. B.01; Gaussian Inc., 2016.

[58] Lu T. (2024). A comprehensive
electron wavefunction analysis toolbox for chemists, Multiwfn. J. Chem. Phys..

[59] Lu T., Chen F. (2012). Multiwfn: A multifunctional
wavefunction analyzer. J. Comput. Chem..

[60] Shuai Z. (2020). Thermal Vibration
Correlation Function Formalism for Molecular Excited State Decay Rates. Chin. J. Chem..

