# Supplementary material for: Ligand-Based Redox Chemistry and Anti-Kasha Fluorescence in Silver(I) Tripyrrindione Radical
Source: Inorg Chem. 2026 Feb 4;65(6):3459–67. doi: 10.1021/acs.inorgchem.5c05116 (PMC12914627; doi:10.1021/acs.inorgchem.5c05116)
Supplement: Supplementary file 1 [file ic5c05116_si_001.pdf]

Supporting Information

Ligand-based redox chemistry and anti-Kasha fluorescence in silver(I) tripyrrindione radical

Iva Habenšus,[a] Qi Sun,[a] Andrei V. Astashkin,[a] Lily J. North,[a]
Jean-Luc Brédas,[a] Veaceslav Coropceanu,[a]* Elisa Tomat [a,b]*

[a] *The University of Arizona, Department of Chemistry and Biochemistry, 1306 E. University Blvd., Tucson AZ 85721-0041, USA*
[b] *University of Vienna, Faculty of Chemistry, Institute of Inorganic Chemistry, Währinger Straße 42, 1090 Wien, Austria*

* coropceanu@arizona.edu; elisa.tomat@univie.ac.at

Contents ***pages***

Chemical characterization data (Figures S1-S11, Tables S1-S2) S2-S7

Computational details (Figures S12-S13, Tables S3-S5) S9-S17

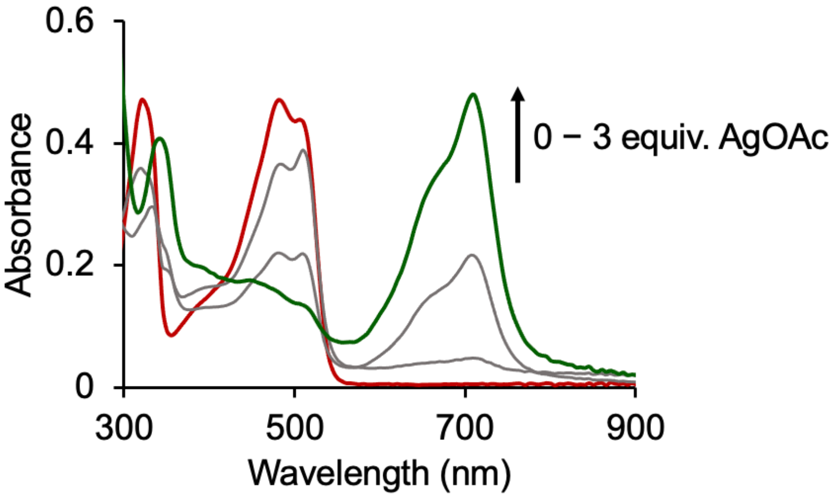

Figure S1. Optical absorption changes upon addition of 1.0, 2.0, or 3.0 equiv. of AgOAc to a solution of H_3TD2 (red trace) in DMSO at room temperature under aerobic conditions.

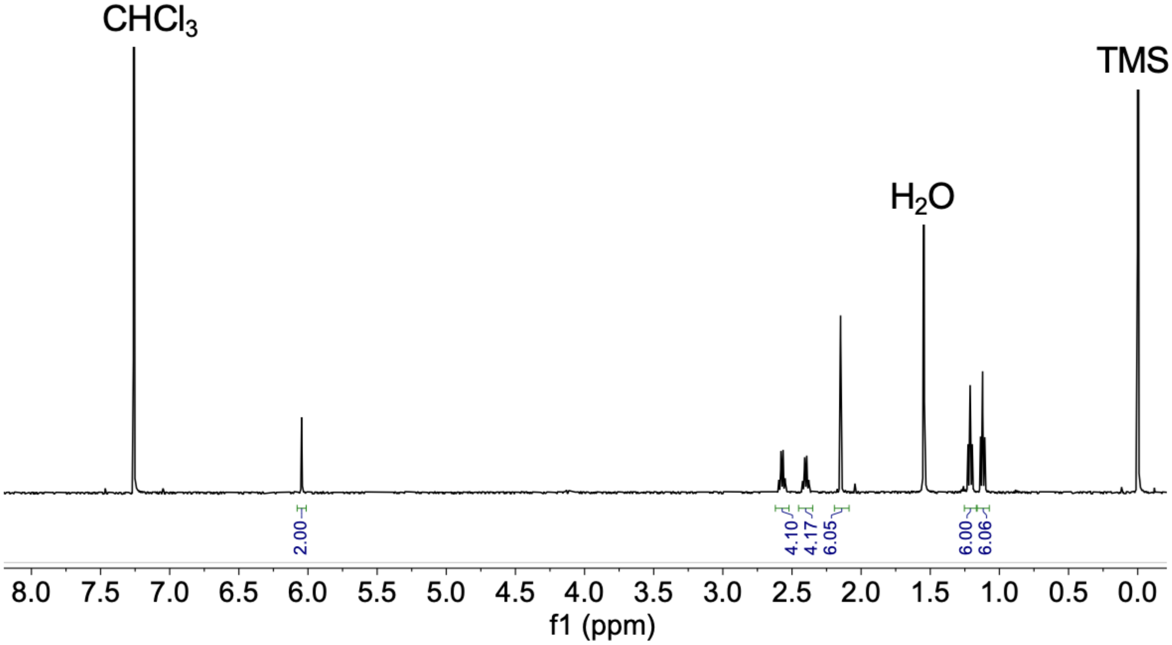

Figure S2. ^1H NMR spectrum of [Ag($TD2_{ox}$)] (500 MHz, $CDCl_3$).

Table S1. Crystallographic details for [Ag(TD2$_{ox}$)] and [CoCp$_2$][Ag(TD2$^•$)].

	**[Ag(TD2$_{ox}$)]**	**[CoCp$_2$][Ag(TD2$^•$)]**
Molecular Formula	$C_{24}H_{28}N_3O_2Ag$	$C_{34}H_{38}N_3O_2CoAg$
Formula Weight [g·mol^{-1}]	498.36	687.47
Temperature [K]	100	100
Crystal Class	Monoclinic	Triclinic
Space Group	C2/c	P-1
a [Å]	20.432(5)	8.5879(6)
b [Å]	10.631(2)	10.6564(6)
c [Å]	20.417(4)	17.0972(10)
α [°]	90	74.630(2)
β [°]	103.619(9)	78.910(2)
γ [°]	90	83.495(2)
Volume [Å^3]	4309.9(15)	1477.32(16)
Z	8	2
ρ_{calc} [g·cm^3]	1.536	1.545
μ [mm^{-1}]	0.961	1.260
F(000)	2048.0	706.0
Crystal Size [mm]	0.11 x 0.03 x 0.016	0.1 x 0.1 x 0.03
Measured Reflections	80412	55210
Independent Reflections, I > 2σ[*I*]	4427	6267
R_{int}	0.2150	0.1031
Goodness-of-fit on F^2	1.024	1.087
R_1, $I > 2\sigma[I]$	0.0513	0.0535
wR_2, all data	0.1320	0.1341
Peak/hole	1.21/-0.91	1.27/-0.99
CCDC Number	2350553	2350552

$R_1 = \Sigma[|F_o| - |F_c|]/\Sigma|F_o|$ $wR_2 = [\Sigma w(F_o^2 - F_c^2)/\Sigma wF_o^4]^{1/2}$

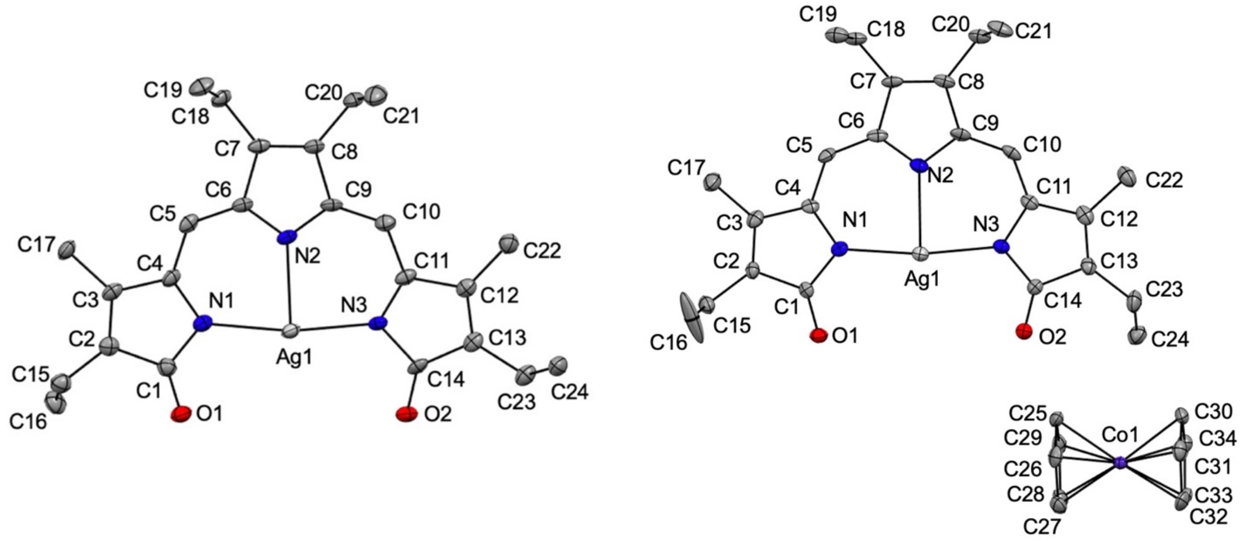

Figure S3. Full labeling scheme for [Ag(TD2$_{ox}$)] (left) and [CoCp$_2$][Ag(TD2$^{\bullet}$)] (right). Carbon-bound hydrogens in calculated positions are omitted for clarity. Atoms are displayed as thermal ellipsoids set at 50% probability level.

Table S2. Comparison of selected bond lengths in Ag(I) and Pt(II) tripyrrindione complexes.

	[Ag^{I}(TD2$_{ox}$)]	[Ag^{I}(TD2$^{\bullet}$)]$^{-}$	[Pt^{II}(TD2$_{ox}$)(H$_2$O)]$^{+}$	[Pt^{II}(TD2$^{\bullet}$)(H$_2$O)]
M1-N1	2.147(4)	2.124(4)	1.992(4)	1.997(3)
M1-N2	2.310(4)	2.330(4)	1.970(4)	1.971(3)
M1-N3	2.153(4)	2.124(4)	2.003(4)	2.004(3)
M1-O3	---	---	2.045(3)	2.037(2)
N1-C1	1.429(6)	1.383(6)	1.450(6)	1.384(4)
N1-C4	1.330(6)	1.354(6)	1.336(6)	1.381(4)
N2-C6	1.348(7)	1.345(7)	1.378(6)	1.373(4)
N2-C9	1.339(6)	1.350(7)	1.371(6)	1.368(4)
N3-C11	1.350(6)	1.381(6)	1.336(6)	1.378(4)
N3-C14	1.402(7)	1.382(6)	1.437(6)	1.382(4)
O1-C1	1.212(6)	1.233(6)	1.206(5)	1.243(4)
O2-C14	1.210(6)	1.210(6)	1.210(5)	1.247(4)
C1-C2	1.487(7)	1.492(7)	1.478(7)	1.472(4)
C2-C3	1.334(7)	1.342(7)	1.333(7)	1.347(5)
C3-C4	1.505(7)	1.475(7)	1.485(7)	1.467(4)
C4-C5	1.410(7)	1.399(7)	1.395(7)	1.373(5)
C5-C6	1.380(7)	1.409(8)	1.367(7)	1.391(4)
C6-C7	1.489(6)	1.466(7)	1.458(7)	1.448(5)
C7-C8	1.346(8)	1.354(8)	1.352(7)	1.357(4)
C8-C9	1.499(7)	1.464(7)	1.468(7)	1.437(5)
C9-C10	1.389(8)	1.413(8)	1.376(7)	1.400(4)
C10-C11	1.415(7)	1.380(7)	1.387(7)	1.367(4)
C11-C12	1.483(7)	1.482(7)	1.488(7)	1.466(4)
C12-C13	1.345(7)	1.340(8)	1.332(7)	1.346(5)
C13-C14	1.495(7)	1.506(7)	1.481(7)	1.472(4)

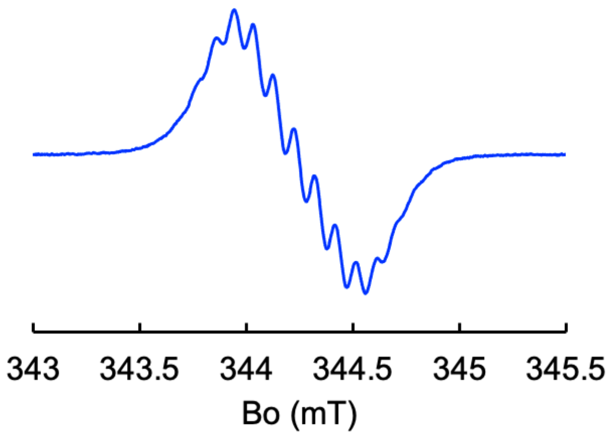

Figure S4. EPR spectrum of [Pd(TD2$^{\bullet}$)(H_2O)] (250 μM) in toluene at room temperature (298 K). The sample was prepared in a glovebox under nitrogen atmosphere. Experimental conditions: mw frequency, 9.650 GHz; mw power, 2 mW; field modulation amplitude, 0.02 mT.

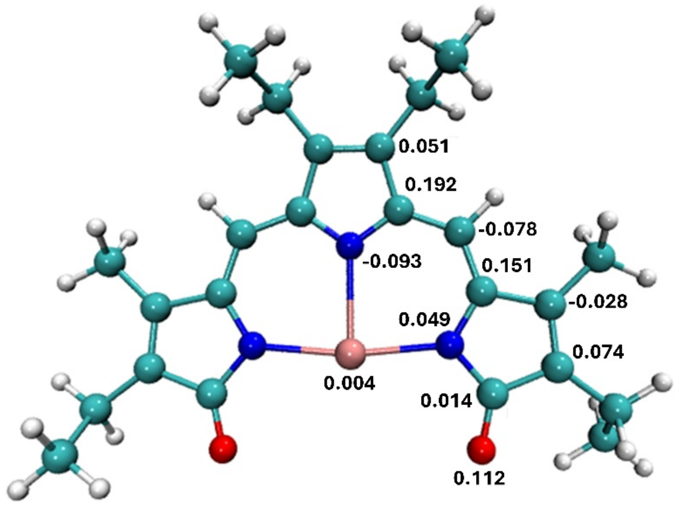

Figure S5. Spin density distribution in [Ag(TD2$^{\bullet}$)]$^{-}$ computed at the ωB97X-D/def2-SVP level. The spin densities are identical in symmetry-related positions.

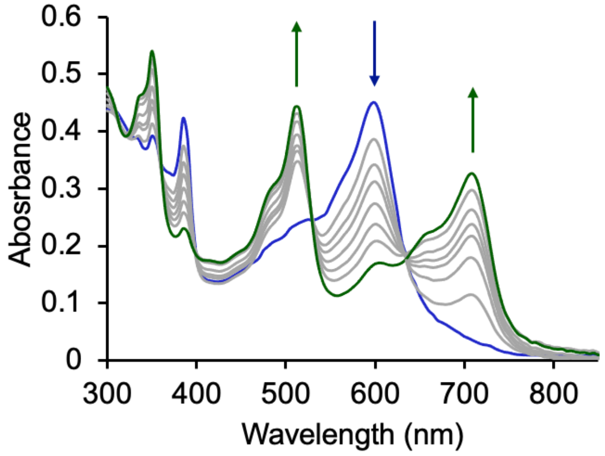

Figure S6. Optical spectral changes upon exposure to air of a solution of [$CoCp_2$][Ag(TD2$^{\bullet}$)] (blue trace at t = 0 min) in dichloromethane at room temperature. The appearance of several new bands is indicative of degradation, including partial reoxidation of the ligand, over a period of ~7 min (green trace at t = 7 min).

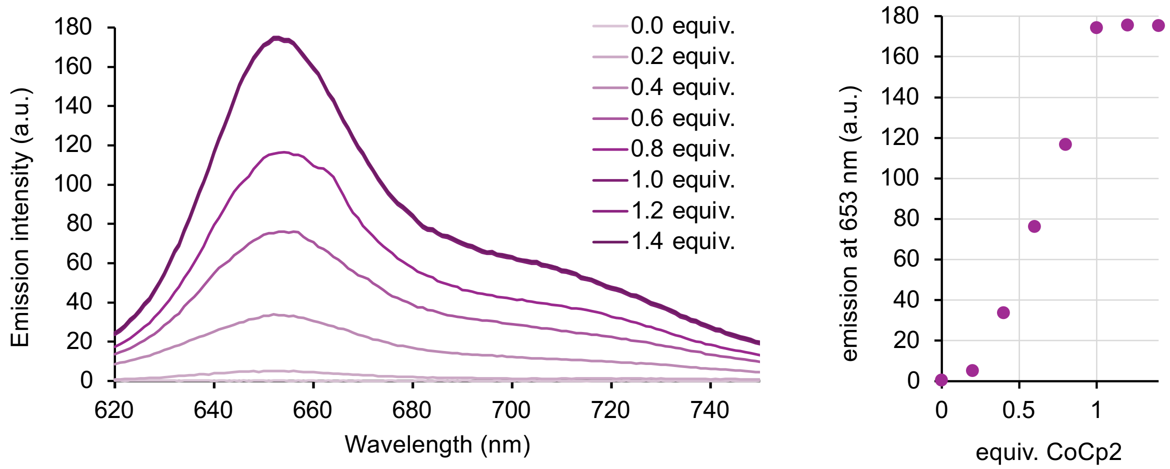

Figure S7. Fluorescence emission spectra (λ_{ex} = 605 nm) observed upon reduction of [Ag(TD2$_{ox}$)] (50 μM) with increasing amounts of $CoCp_2$ (0.0-1.4 equiv.) in THF at room temperature under a nitrogen atmosphere.

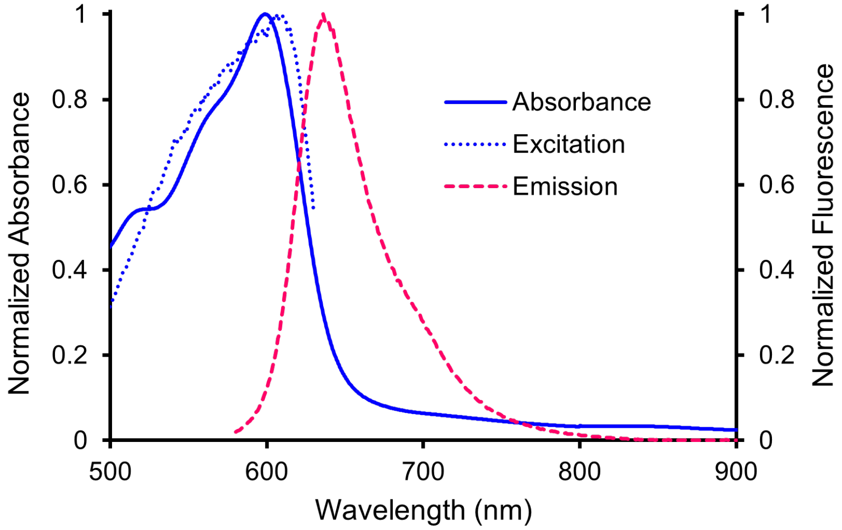

Figure S8. Normalized absorbance, excitation and emission spectra of [CoCp$_2$][Ag(TD2$^•$)] (100 μM in THF).

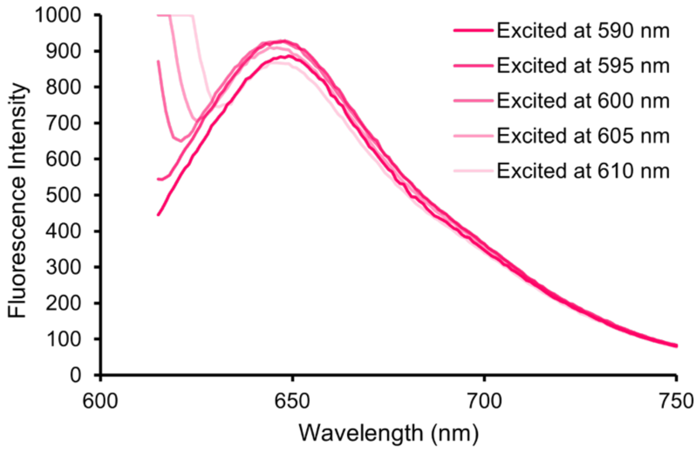

Figure S9. Emission spectra of [CoCp$_2$][Ag(TD2$^•$)] (100 μM in THF) at different excitation wavelengths (590, 595, 600, 605, 610 nm).

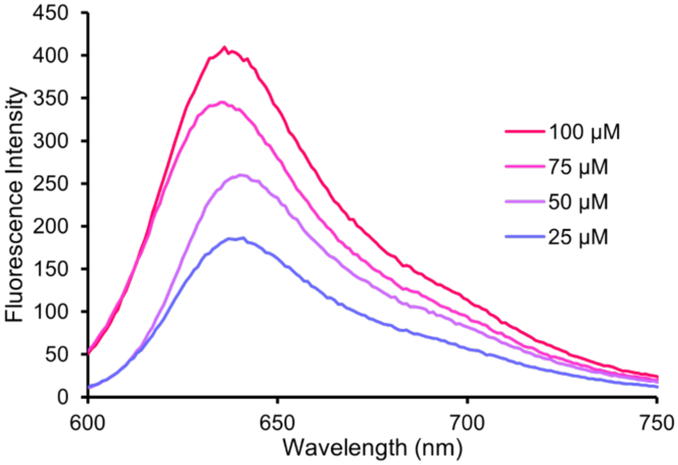

Figure S10. Emission spectra of [CoCp$_2$][Ag(TD2$^\bullet$)] at varying concentrations (100, 75, 50, 25 µM) in THF.

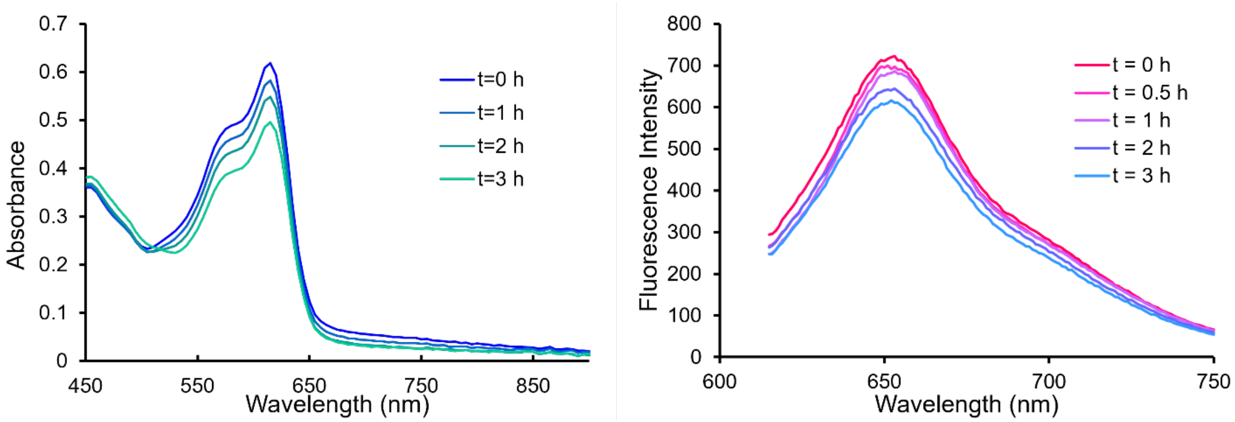

Figure S11. Absorbance (left, 35 µM in THF) and emission (right, 75 µM in THF) spectra of [CoCp$_2$][Ag(TD2$^\bullet$)] monitored over a period of 3 h.

Table S3. Selected bond lengths (Å) in Ag(I) tripyrrindione complexes, as obtained at the DFT/ ωB97X-D/def2-SVP optimized ground-state geometry.

	$[Ag^{I}(TD2_{ox})]$	$[Ag^{I}(TD2^{\bullet})]^{-}$
Ag-N1	2.176	2.160
Ag-N2	2.367	2.363
Ag-N3	2.176	2.160

Table S4. Selected bond lengths (Å) in $[Ag(TD2_{ox})]$ and $[Ag(TD2^{\bullet})]^{-}$ as obtained at the TD-DFT/ ωB97X-D/def2-SVP optimized excited-state geometries (S_1, D_1, D_2, and D_3).

	**S_1**	**D_1**	**D_2**	**D_3**
Ag-N1	2.060	2.049	2.170	2.171
Ag-N2	2.130	2.132	2.371	2.385
Ag-N3	2.059	2.049	2.179	2.174

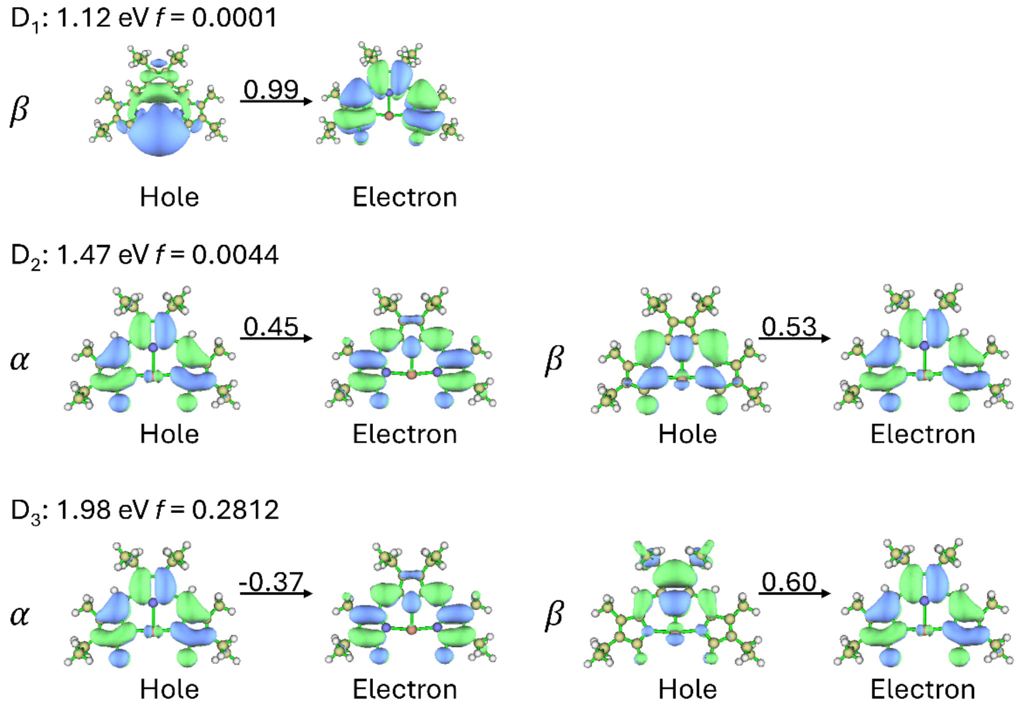

Figure S12. NTOs for transitions from D_0 to the D_1, D_2, and D_3 excited states in $[Ag(TD2^{\bullet})]^{-}$, as computed in the D_3 geometry at the TD-DFT/ωB97X-D/def2-SVP level.

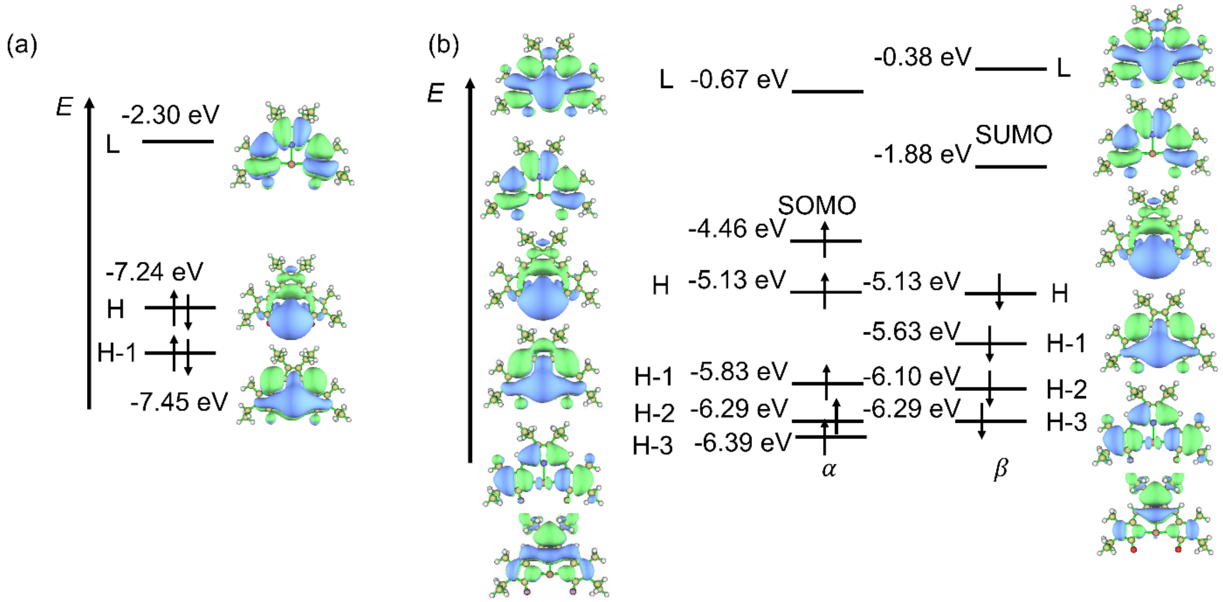

Figure S13. Molecular orbitals and their energies for (a) [Ag(TD2$_{ox}$)] and (b) [Ag(TD2$^{•}$)]$^{-}$, as computed in the ground-state geometry at the TD-DFT/ωB97X-D/def2-SVP level.

Table S5. Cartesian coordinate parameters in the optimized geometries of the investigated molecules. The DFT and TD-DFT calculations were performed at the ωB97X-D/def2-SVP level.

[Ag(TD2$_{ox}$)]			
S_0 geometry			
Ag	-0.013580	-1.291146	-0.000876
O	2.754378	-3.302046	0.016351
O	-2.803066	-3.238852	0.346126
N	2.146594	-1.074838	-0.146991
N	-0.000130	1.066837	-0.212001
N	-2.170911	-1.025996	0.101084
C	4.331288	-0.260001	-0.339572
C	-2.395244	1.431724	-0.105842
H	-3.163974	2.204290	-0.133487
C	3.060998	-2.138586	-0.103342
C	-1.078347	1.867117	-0.211950
C	-2.866195	0.107681	0.039245
C	2.399137	1.377826	-0.380400
H	3.176452	2.133548	-0.494974
C	-0.660374	3.296302	-0.348205
C	-4.345598	-0.165010	0.149327
C	1.088624	1.842460	-0.336652
C	-3.096907	-2.069722	0.253338
C	0.690285	3.280941	-0.426252
C	-4.485882	-1.493611	0.285497
C	2.854706	0.043915	-0.287394
C	4.456699	-1.592686	-0.232107
C	1.644871	4.429548	-0.511772
H	1.119708	5.308619	-0.914845
H	2.442172	4.191110	-1.234332
C	-5.392538	0.895382	0.103610
H	-5.250027	1.630115	0.911198
H	-6.396492	0.464092	0.206843
H	-5.355070	1.446015	-0.849123
C	-5.698726	-2.354872	0.398746
H	-6.547748	-1.757298	0.763448
H	-5.503604	-3.131090	1.155708
C	-2.044382	4.834765	1.093693
H	-2.734585	5.690597	1.072112
H	-1.182027	5.104956	1.720915
H	-2.560644	3.993178	1.579842
C	5.659174	-2.474084	-0.181552
H	6.522653	-1.952773	-0.621391
H	5.466539	-3.360174	-0.807272
C	-1.591675	4.466732	-0.323724
H	-2.473513	4.247539	-0.947384
H	-1.097033	5.333600	-0.787287
C	5.389821	0.780599	-0.478739
H	5.260036	1.358616	-1.406785
H	6.389486	0.327871	-0.495039
H	5.353357	1.494867	0.358484

C	-6.062994	-3.026434	-0.929432
H	-6.292459	-2.274810	-1.699596
H	-6.943111	-3.674679	-0.808919
H	-5.230822	-3.646111	-1.294520
C	5.989991	-2.926959	1.244274
H	6.215550	-2.063752	1.888279
H	6.863229	-3.595372	1.247318
H	5.142488	-3.471055	1.686322
C	2.269095	4.783920	0.842528
H	1.492723	5.073120	1.566182
H	2.972249	5.623130	0.739655
H	2.818425	3.929187	1.265401

$[Ag(TD2^{\bullet})]^{-}$

D_0 geometry

Ag	0.010770	-1.251983	0.000435
O	2.772925	-3.265259	0.369793
O	-2.736576	-3.327315	0.039866
N	-2.138862	-1.097305	-0.142529
N	2.159209	-1.048556	0.102866
N	0.003533	1.097113	-0.258298
C	-4.421367	-1.606919	-0.229627
C	-3.027030	-2.142208	-0.093140
C	2.872263	0.117667	0.024926
C	-2.860623	0.056214	-0.297138
C	-0.698477	3.283748	-0.427505
C	0.671783	3.298888	-0.350877
C	-4.314270	-0.267083	-0.347445
C	1.083461	1.897131	-0.240584
C	-1.088940	1.873067	-0.362127
C	2.414452	1.420326	-0.126041
H	3.191381	2.187660	-0.151242
C	4.328229	-0.178533	0.134988
C	3.055345	-2.076009	0.260503
C	5.408549	0.852922	0.079236
H	5.397427	1.390820	-0.881753
H	6.400195	0.395319	0.196590
H	5.286279	1.606606	0.872996
C	-5.619532	-2.497203	-0.177943
H	-6.494370	-1.981972	-0.604872
H	-5.426087	-3.377035	-0.813159
C	6.023935	-3.061450	-0.920227
H	6.895891	-3.721825	-0.798129
H	6.266386	-2.311669	-1.688947
H	5.185012	-3.668465	-1.291274
C	2.027591	4.849401	1.116112
H	2.545120	4.006741	1.599853

H	1.156309	5.104272	1.738304
H	2.710507	5.712615	1.112740
C	1.591112	4.482366	-0.306248
H	1.096083	5.350202	-0.770288
H	2.485269	4.280310	-0.919314
C	-2.414257	1.367756	-0.395891
H	-3.199059	2.119422	-0.505985
C	4.445683	-1.514370	0.284281
C	-1.642644	4.446758	-0.489194
H	-2.458512	4.224407	-1.197105
H	-1.118179	5.324769	-0.898548
C	5.652369	-2.386714	0.403914
H	5.443848	-3.165269	1.155527
H	6.507762	-1.801981	0.777770
C	-5.401546	0.747575	-0.497555
H	-6.390341	0.269751	-0.518090
H	-5.285272	1.326795	-1.426986
H	-5.392908	1.467764	0.335646
C	-2.242089	4.805883	0.874511
H	-2.791106	3.952523	1.301455
H	-2.939328	5.653594	0.792339
H	-1.450764	5.081052	1.588106
C	-5.935508	-2.972494	1.243539
H	-6.799212	-3.654543	1.251397
H	-6.165174	-2.120364	1.901557
H	-5.072241	-3.506754	1.666620

D_1 geometry

Ag	-0.00606731	-0.89023478	-0.00433135
O	-2.44379471	-3.14843327	-0.37237133
O	2.39777500	-3.20688517	-0.02374486
N	2.03691433	-0.92760754	0.14918382
N	-2.04995554	-0.87811163	-0.14889370
N	0.00595584	1.22911708	0.22849886
C	4.23500414	-1.63735606	0.24334936
C	2.80243040	-2.05766010	0.10610259
C	-2.85585327	0.24549299	-0.06900295
C	2.85527282	0.17824396	0.31038189
C	0.72300756	3.34824817	0.42535537
C	-0.68320899	3.36425244	0.32385288
C	4.25547306	-0.29128333	0.36402927
C	-1.09537558	2.02000387	0.20241059
C	1.11837196	1.99480345	0.35716618
C	-2.44696808	1.53145916	0.08616588
H	-3.22880955	2.29376549	0.12647053
C	-4.26388868	-0.19137880	-0.16832044
C	-2.83168860	-1.98969778	-0.28438236
C	-5.43045490	0.74276538	-0.11692945
H	-5.44809917	1.30067596	0.83295410
H	-6.38290112	0.20189320	-0.20933284

H	-5.37836150	1.48791527	-0.92685551
C	5.33926329	-2.64270975	0.20262528
H	6.28064234	-2.19575406	0.56335524
H	5.08503071	-3.45996747	0.89836186
C	-5.60370681	-3.27832983	0.91048576
H	-6.39989866	-4.03306658	0.80784273
H	-5.88492496	-2.58578039	1.71926705
H	-4.67677426	-3.79103057	1.20620616
C	-1.99749324	4.98899998	-1.11589843
H	-2.53181670	4.17101466	-1.62329631
H	-1.11234826	5.22556635	-1.72629394
H	-2.65297876	5.87589205	-1.10377985
C	-1.58450276	4.56573311	0.29764029
H	-1.08520744	5.41330471	0.79698535
H	-2.49334527	4.36825059	0.89237389
C	2.46243588	1.47584216	0.39921039
H	3.25200078	2.22098666	0.52268305
C	-4.26080147	-1.53615418	-0.30151646
C	1.64278108	4.53310058	0.50166027
H	2.50324260	4.30071813	1.15226914
H	1.12257949	5.37302470	0.99300033
C	-5.37799692	-2.52235323	-0.40248513
H	-5.11984506	-3.25295870	-1.18681017
H	-6.30847990	-2.02224029	-0.71912844
C	5.43240873	0.61910338	0.51224630
H	6.37602948	0.05558905	0.52797876
H	5.36966581	1.20528271	1.44295783
H	5.48218671	1.33929154	-0.32004827
C	2.15926510	4.99518053	-0.86483264
H	2.70796332	4.18206575	-1.36474790
H	2.83204392	5.86469095	-0.77741865
H	1.32226567	5.27402194	-1.52341079
C	5.53548114	-3.23720014	-1.19499053
H	6.32772133	-4.00294807	-1.19876883
H	5.80910119	-2.45514731	-1.92059781
H	4.59882806	-3.70385479	-1.53268997

D_2 geometry

Ag	-0.00832307	-1.27497064	-0.04195069
O	-2.77838579	-3.26002094	-0.40032156
O	2.73918575	-3.30955210	-0.00415720
N	2.14793913	-1.06864257	0.17341014
N	-2.16735273	-1.02825334	-0.17000869
N	-0.00103872	1.10071026	0.19620720
C	4.42731813	-1.59403792	0.23890028
C	3.04422443	-2.13117464	0.12126631
C	-2.87838147	0.11546947	-0.06578136
C	2.86666863	0.06719313	0.30495176
C	0.68643670	3.29226828	0.42779061
C	-0.67744536	3.30327947	0.34131861

C	4.31243105	-0.24640468	0.34757475
C	-1.08806750	1.89579758	0.19576538
C	1.08958941	1.87811531	0.33644520
C	-2.40361670	1.43800425	0.08379416
H	-3.17955031	2.20565018	0.13105488
C	-4.32764314	-0.17800668	-0.12346283
C	-3.07283539	-2.07744586	-0.29351263
C	-5.40485887	0.85379235	-0.02379771
H	-5.36462707	1.38777427	0.94031890
H	-6.40098218	0.39648770	-0.11154514
H	-5.31021682	1.61584695	-0.81531444
C	5.62623392	-2.48400727	0.19442405
H	6.52235658	-1.93634637	0.53127195
H	5.47117257	-3.31075011	0.90826289
C	-5.91752953	-3.12792046	0.99449188
H	-6.79273602	-3.79402101	0.92415060
H	-6.09659106	-2.40893541	1.80924049
H	-5.04091904	-3.73566660	1.26224607
C	-2.04537704	4.86464871	-1.09933604
H	-2.55188142	4.01911252	-1.58860259
H	-1.17608085	5.12927149	-1.72061566
H	-2.73546028	5.72362878	-1.08899311
C	-1.60124720	4.48267598	0.31684728
H	-1.11129564	5.34912025	0.79117201
H	-2.49219622	4.26544724	0.93061194
C	2.40122189	1.39863140	0.39037085
H	3.18018297	2.15484689	0.51280786
C	-4.45276343	-1.52116680	-0.26726173
C	1.62634469	4.45522044	0.52194720
H	2.42992447	4.22121608	1.24099792
H	1.09717406	5.32997077	0.93484940
C	-5.65881460	-2.39968546	-0.32855983
H	-5.49886760	-3.15269000	-1.11797686
H	-6.54724006	-1.81286132	-0.61686305
C	5.39502990	0.77625418	0.47885431
H	6.38848308	0.30519467	0.46783560
H	5.30340219	1.34815008	1.41735926
H	5.36012584	1.50689343	-0.34638990
C	2.25126205	4.82980768	-0.82647290
H	2.78837721	3.97106564	-1.25681428
H	2.96089311	5.66649106	-0.72424860
H	1.47310323	5.12310669	-1.54762206
C	5.86521146	-3.08107816	-1.19601317
H	6.73561271	-3.75705881	-1.19865600
H	6.04426205	-2.28843627	-1.93934552
H	4.98000533	-3.65201299	-1.51138253

D_3 geometry

Ag	0.012613	-1.236729	-0.000941
O	2.759686	-3.270446	0.341406

O	-2.715927	-3.333957	0.026944
N	-2.153455	-1.091491	-0.145959
N	2.177814	-1.039472	0.097784
N	0.001947	1.103449	-0.222469
C	-4.428488	-1.633735	-0.229082
C	-3.046033	-2.158847	-0.099792
C	2.895333	0.109899	0.036701
C	-2.882024	0.044922	-0.288243
C	-0.707027	3.273622	-0.425816
C	0.677603	3.290381	-0.347115
C	-4.324333	-0.283572	-0.337597
C	1.087068	1.910137	-0.219102
C	-1.095255	1.884059	-0.342888
C	2.415368	1.427454	-0.106171
H	3.194302	2.193935	-0.129204
C	4.339898	-0.190091	0.147846
C	3.080658	-2.089827	0.250512
C	5.420878	0.840326	0.106809
H	5.411818	1.393739	-0.846213
H	6.412491	0.380840	0.217807
H	5.300532	1.584427	0.910880
C	-5.628216	-2.523221	-0.178659
H	-6.501159	-2.004677	-0.605931
H	-5.440448	-3.402920	-0.816428
C	6.049029	-3.070331	-0.925491
H	6.923373	-3.728100	-0.804365
H	6.292090	-2.313179	-1.686773
H	5.215048	-3.678097	-1.306896
C	1.993869	4.906505	1.093592
H	2.511865	4.086238	1.614397
H	1.108340	5.169825	1.692054
H	2.666549	5.778178	1.075924
C	1.587217	4.484079	-0.322179
H	1.096554	5.332890	-0.825701
H	2.496588	4.273362	-0.910356
C	-2.416846	1.370754	-0.379225
H	-3.204539	2.120465	-0.490408
C	4.456995	-1.536895	0.286524
C	-1.642473	4.444368	-0.512104
H	-2.462149	4.216162	-1.214657
H	-1.112353	5.309186	-0.943071
C	5.666183	-2.407464	0.401693
H	5.461650	-3.193417	1.147286
H	6.517631	-1.822418	0.784486
C	-5.414756	0.728178	-0.477582
H	-6.402581	0.248151	-0.498581
H	-5.304278	1.315677	-1.403355
H	-5.407029	1.444467	0.359920
C	-2.240459	4.844760	0.840590
H	-2.801760	4.008132	1.284983
H	-2.926230	5.700428	0.739983

H	-1.447091	5.123708	1.550688
C	-5.950733	-3.001074	1.240748
H	-6.816738	-3.680587	1.245172
H	-6.179246	-2.149458	1.899889
H	-5.090971	-3.539191	1.666551
